# Human *CPTP* promotes growth and metastasis via sphingolipid metabolite ceramide and PI4KA/AKT signaling in pancreatic cancer cells

**DOI:** 10.7150/ijbs.70007

**Published:** 2022-07-27

**Authors:** Yanqun Zhang, Shenying Ji, Xiangyu Zhang, Mengyun Lu, Yihong Hu, Yucheng Han, Guanghou Shui, Sin Man Lam, Xianqiong Zou

**Affiliations:** 1 Department of Oncology, Xiangya Hospital, Central South University, Changsha 410008, Hunan, P. R. China; 2 Affiliated Stomatology Hospital of Guilin Medical University, Guilin 541004, Guangxi, P. R. China; 3 School of Basic Medical Sciences, Guilin Medical University, Guilin 541100, Guangxi, P. R. China; 4 State Key Laboratory of Molecular Developmental Biology, Institute of Genetics and Developmental Biology, Chinese Academy of Sciences, Beijing 100101, P. R. China; 5 State Key Laboratory of Molecular Developmental Biology, Institute of Genetics and Developmental Biology, Chinese Academy of Sciences, Beijing 100101, P. R. China; LipidALL Technologies Company Limited, Changzhou 213022, Jiangsu, P. R. China

**Keywords:** *CPTP*, metastasis, epithelial-mesenchymal transition, sphingolipid metabolite, pancreatic cancer

## Abstract

Pancreatic cancer (PC) is a devastating solid malignancy with a dismal prognosis. The treatment of metastatic PC is a current challenge for medical oncologists due to a lack of early detection, drug resistance, and relapse. Therefore, potential biomarkers and effective therapeutic targets for PC are urgently required. Ceramide-1-phosphate transfer protein (CPTP) is a member of the glycolipid transfer protein family, which is associated with autophagy and inflammation regulation. The roles and mechanisms of *CPTP* in PC have not been clarified. In this study, by RT-qPCR and immunohistochemistry analysis, we found that *CPTP* is highly expressed in PC and is associated with a poor prognosis in PC patients. By using cell counting kit-8, colony formation, transwell and matrigel assays* in vitro*, as well as xenograft model assays *in vivo*, we further proved that *CPTP* enhanced PC cells growth and metastasis. In PC cells, human *CPTP* promotes growth and metastasis via sphingolipid metabolite ceramide and PI4KA/AKT signaling. Sp (specific protein)-1 and Sp3 transcription factors also act as upstream positive regulators of *CPTP* expression in PC cells. Collectively, these findings suggested that *CPTP* may function as a pro-tumorigenic gene in PC cells and could be a promising therapeutic target in PC.

## Introduction

Pancreatic cancer (PC) is one of the most invasive malignant tumors, and has a dismal prognosis. Pancreatic ductal adenocarcinoma accounts for >85% of all PC cases 1, 2. According to the latest cancer statistics (2021), PC is the fourth most common cause of cancer death in the United States 3. Despite decades of efforts to improve treatment, the mortality rate has remained largely unchanged, with a 5-year survival rate lower than 9% 4, 5. PC has a high propensity to metastasize, as the pancreas is rich in blood and lymphatic vessels, with incomplete capsules, resulting in tumors metastasizing to the abdominal cavity, lymph nodes and liver 6, 7. The main therapies currently include surgery, chemo-/radiotherapy, immunotherapy and targeted drugs, such as inhibitors for vascular endothelial growth factor or EGFR 8-12. Early detection is important for cancer diagnosis; however, as effective screening methods for PC are limited, most patients are diagnosed in the mid-late stage of the disease 13-15. In addition, high metastatic potential and resistance to chemotherapy result in poor patient outcomes 16, 17. Therefore, in order to identify effective therapeutic targets in PC to improve clinical outcomes, an improved understanding into molecular carcinogenesis and the identification of novel early diagnostic biomarkers of PC are urgently required.

Metastasis is associated with a poor prognosis and is the primary cause of patient mortality 18. Epithelial-mesenchymal transition (EMT) is a key factor in tumor metastasis 19. EMT is a gradual process, in which partial cells not only retain some characteristics of epithelial cells (such as expression alteration of E-cadherin, β-catenin, zonula occludens protein 1, desmoplakin and claudin-1), but also obtain markers of mesenchymal cells (including expression alteration of vimentin, snail, slug, fibronectin, twist and ZEB1/2), while other parts of the cells become fully mesenchymal cells 20. These cells are usually localized to the periphery of the tumor, acquiring local and systemic invasive abilities via EMT, and eventually lead to tumor metastasis 20, 21. Several signaling pathways participate in this process, including the PI3K/AKT, Wnt, Ras/Raf/MEK/ERK and Notch signaling, and complex crosstalk between them exists 22-25.

Ceramide-1-phosphate transfer protein (CPTP; also known as GLTPD1) belongs to the glycolipid transfer protein (GLTP) family 26. *CPTP* consists of 3 exons separated by 2 introns and is located at 1p36.33. It is also the only currently identified protein that can selectively transport ceramide-1-phosphate (C1P) in mammals 27, 28. C1P is involved in a range of biological functions, including cell proliferation and migration/invasion 29, 30. IVA phospholipase A2 (IVAcPLA2) is also activated, which translocates to the Golgi apparatus to promote arachidonic acid and the downstream products, pro-inflammatory eicosanoids, are signaling molecules for inflammation and cancer 27, induction of autophagy 31, and they elevate the levels of pro-inflammatory cytokines in cells 27. Besides, C1P also plays a significant role in sphingolipid regulation 26, 32, suggesting that the C1P transport protein, CPTP, is crucial for the development of diseases associated with these processes. Bioinformatics analysis has revealed the abnormal expression levels of *CPTP* in several tumors, such as PC, lymphoid neoplasm diffuse large B-cell lymphoma and ovarian serous cystadenocarcinoma 33. The expression level of *CPTP* is low in patients with severe acute pancreatitis (SAP) 34; therefore, it is possible that *CPTP* is involved in the molecular mechanisms to effect tight junction proteins by downregulating the expression levels of IVAcPLA2; thus, providing protection to patients with SAP 34. On the other hand, *CPTP* is a direct target of the tumor suppressor, microRNA-328 35, suggesting that *CPTP* may be associated with tumorigenesis. Furthermore, *CPTP* knockdown, induced by small interfering (si)RNA results in autophagy and pro-inflammatory cytokine IL-1β and IL-18 release depend on the NLR family pyrin domain containing 3 inflammasome-based mechanism 36. In addition, inflammasome assembly is autophagy-dependent 36. Another study demonstrated that *CPTP* is associated with breast and colon tumor progression 37; however, the role, underlying mechanism, transcriptional regulation and disease-relevant clinical research of *CPTP* in cancer have not been reported.

The aim of this study was to investigate the functions of *CPTP*, including in PC cells proliferation, migration and invasion *in vitro,* and the tumorigenic ability *in vivo*, as well as the mechanism underlying PC initiation and progression. In addition, the association of the transcription factors, specific protein (Sp)-1 and Sp-3 in the upregulation of *CPTP* expression in PC cells was also investigated. The present study may provide new insights into the diagnosis and treatment in patients with PC.

## Material and methods

### Cell culture and stable cell line construction

Human PANC-1 and MIA PaCa-2 (American Type Culture Collection) cell lines were cultured in DMEM (Gibco) with 10% FBS (Sigma-Aldrich; Merck KGaA) and incubated at 37˚C in 5% CO_2_. The human *CPTP* DNA sequences were amplified using PCR and advantage GC genomic LA polymerase (Thermo Fisher Scientific, Inc.), and cloned into the pFLAG-CMV4 plasmid (Sigma-Aldrich; Merck KGaA). The short hairpin RNAs (shRNA) targeting *CPTP* were constructed utilizing the pSuper puro-eGFP plasmid (OligoEngine) 27. The PANC-1 and MIA PaCa-2 cells were transfected with the target plasmids for 48 h with Lipofectamine^®^ 3000 reagent (Invitrogen) referenced to manufacturer's instructions. Subsequently, the cells were selected for 14 days using G418 (Amresco), then single cell clones were obtained using a limiting dilution method. The overexpression and interference efficiencies were detected by Western blot analysis. GSK-A1 or mithramycin A (GLPBio Technology) was used as treatments.

### Bioinformatics analysis

The relationship between *CPTP* expression level and tumor grade was analyzed using the LinkedOmics website 38. The associations between *CPTP* expression level and overall survival (OS) and disease-free survival (DFS) times of PC patients were characterized via GEPIA website which contains high-throughput RNA-sequencing data from The Cancer Genome Atlas (TCGA) and GTEx databases 33. The Gene Ontology (GO)/Kyoto Encyclopedia of Genes and Genomes (KEGG) pathway enrichment and protein-protein interaction network analysis were performed and visualized in the Metascape website and STRING 11.5, respectively 39-40.

### Tissue microarray (TMA) immunohistochemistry (IHC)

PC specimens and adjacent normal tissues were used to construct a TMA (Shanghai Outdo Biological Technology, Ethics Committee approval no. YB M-05-02). The TMA sections were heated at 63˚C for 1 h, then dewaxed in xylene and rehydrated to water in decreasing alcohol gradient. Heat-induced antigen retrieval was carried out by EDTA antigen retrieval agent (cat. no. K8002; Dako; Agilent Technologies, Inc.). Then the TMA sections were washed three times in PBST and incubated with a specific antibody at 4˚C overnight. Anti-CPTP (cat. No. HPA056832; 1:20 dilution; Atlas Antibodies, AB), anti-Sp1 (cat. No. sc-17924X; 1:100 dilution) and anti-Sp3 (cat. No. sc-28305X; 1:40000 dilution) antibodies from Santa Cruz Biotechnology were used. After incubation, the TMA sections were washed with PBST again, and incubated with anti-rabbit HRP secondary antibody (cat. no. EM35111-01, 1:200 dilution; EMAR Biotechnology). The dye, 3,3'-diaminobenzidine was used for color development after the samples were washed three times (1 min each time) with PBST. Finally, the sections were counterstained with hematoxylin, immersed in 0.25% alcohol hydrochloric acid solution for 2 sec, washed with running water and sealed after drying at room temperature (25˚C). An Aperio XT slide scanner (Lecia Microsystems, Inc., USA) was used to scan the TMA image. IHC results were analyzed to determine the score, which included the intensity of IHC staining (1, weak; 2, moderate; 3, strong) and multiplying the percentage of positive cells. Pearson's χ^2^ test was used for correlation analysis. P<0.05 was considered as a statistically significant difference.

### RNA extraction and RT-qPCR

Total RNA of clinical samples which collected from Affiliated Hospital of Guilin Medical University (approval no. QTLL202150), and cells were extracted by TRIzol® (Invitrogen). Total RNA was reverse transcribed then analyzed using semi-quantitative and qPCR. Briefly, 1 μg total RNA was reverse transcribed into cDNA using a FastKing cDNA transcription kit according to the manufacturer's instructions (Tiangen Biotech (Beijing) Co., Ltd.). Semi-qPCR was performed as previously described. SYBR green was used for qPCR and performed at 7500 Fast RT-PCR system (Applied Biosystems) as following thermocycling conditions: initial denaturation for 10 min at 95˚C, 95˚C for 15 sec and 60˚C for 1 min for 40 cycles. The primers for *CPTP* and β-actin are shown in Table S1 27, 41. The RT-qPCR results were analyzed by the 2^-ΔΔCq^ method using β-actin as a reference 42.

### Cell proliferation and colony formation assay

The viability of cells was assessed through CCK-8 (Dongren Chemical Technology Co. Ltd.) assay. 1x10^4^ cells were seeded in 24-well plates per well, and cell proliferation was measured every 24 h until 72 h after complete adherence. A 100 μl CCK-8 reagent was added to the medium and incubated for 2 h at 37˚C. Subsequently, BioTek plate reader was used to detect the cells absorbance at 450 nm and was normalized to that measured on 0 h.

500 cells were seeded in 6-well plates and incubated for 12 days for colony formation assay. The culture medium was changed every two days. Subsequently, the cells were fixed with 10% methanol and stained with 0.2% crystal violet. Colony numbers were measured using Image Pro Plus v6 (Media Cybernetics) and images were then captured. The cell colony formation rate was calculated by the following equation: (cell colony number/500) x 100%.

### Transwell and Matrigel assays

100 μl serum-free cell suspension (contain~3x10^4^ cells) was seeded in the upper chamber of transwell inserts (cat. No. 3422; Corning, Inc.). 750 μl medium with 20% FBS was added to the lower chamber. After cultured for 24 h (invasion for 48 h), the chambers were washed twice with PBS, then the cells on the membranes were fixed with absolute methanol. Cotton swabs were used to remove the cells and stained with Giemsa (Beijing Solarbio Science and Technology) for 30 min. Images of the cells were captured with a Nikon microscope and the numbers of cells were counted.

For invasion assay, the Transwell membrane was covered with Matrigel (Corning, Inc.) at 1:8 dilution and incubated for more than 1h at 37 ˚C. The Matrigel assay was performed as mentioned before.

### Animal experiments

For the tumor formation assay, 5 to 6 weeks male BALB/c-nu mice (Hunan SLAC laboratory animal center) were randomly divided into overexpression or knockdown of *CPTP* and each control group for 6 mice per group. All the mice were subcutaneously injected in the region of the right axilla with 100 μl cell suspension (~2x10^6^/mouse). Tumor measurements were performed every third day, starting two weeks after injection, using a vernier caliper. After 4-6 weeks, the mice were euthanized using CO_2_ (with a volume displacement rate of 30%/min). The following humane endpoints were used: weight loss of >20% or deterioration in health, and a maximum tumor size of 481.263 mm^3^. The tumor specimens of *CPTP* overexpression and control group were removed after 4 weeks of tumor implantation, and the samples from the *CPTP* knockdown and control group were removed after 6 weeks. For lung metastasis assay, the mice were grouped in the same way as in the tumor development experiment (n=5). 5 ×10^6^ CPTP-overexpressing or CPTP-knockdown PANC-1 cells were injected into the mice through tail vein after a week of acclimating. Mice were sacrificed 40 days later, and lung tissues were extracted and stained with hematoxylin and eosin (H&E) for histological analysis. All the animal experiments were consistent with laboratory animal welfare and approved by the Ethics Committee of Guilin Medical University (approval no. 2021-0003; GLMC-IACUC-2022004). Tumor volume was calculated as the following formula: Tumor volume (mm^3^) = (length x width^2^)×0.5.

### RNA sequencing and Label-free proteomic analysis

The samples of the PANC-1 cell line transfected with *CPTP* overexpression plasmid or shRNA were collected and the RNA sequencing assay was performed (Beijing Genomics Institution) as described previously 43. For Label-free proteomic analysis, the samples were prepared using the EasyPep Mini mass spectrometry (MS) Sample Prep kit (cat. no. A40006; Thermo Fisher Scientific, Inc.) reference to the instruction. After treatment, MS-ready peptide was obtained, then dried using vacuum centrifugation. Finally, the peptides were analyzed using a Proxeon EASY-nLC 1000 chromatograph coupled to a Thermo Fisher Q Exactive mass spectrometer (Thermo Fisher Scientific). For proteomic data analysis, the peptides were identified using Proteome Discoverer software v2.4. Trypsin was utilized as the digestion enzyme with a maximum of 2 missed cleavages 44. Cysteine iodine acetylation was adopted as a fixed modification, meanwhile using N-terminal acetylation and methionine oxidation as variable modifications. The peptide mass tolerance was set to 10 ppm for searching. Peptide spectrum matches were accepted with fragment mass tolerance to 0.6 Da and a FDR ≤1.0% 45. The screening criteria of differentially expressed proteins (DEPs) was fold change >1.2 and Abundance Ratio Adj. P-Value<0.05.

### Lipid extraction and lipidomic analysis

Lipids were extracted from ~1x10^6^ cells using improved versions of the Bligh and Dyer method 46. Briefly, added 750 µl chloroform : methanol : MilliQ water (volume ratio, 3:6:1) mix to the cell precipitate for homogenized, then centrifuged at 1500 rpm for 1 h under cryogenic condition. Subsequently, 600 µl deionized water and chloroform mix (volume ratio, 7:5) were added to induce phase separation. The underlying organic phase containing lipids was transferred to a new tube after centrifugation. 450 µl chloroform was added to the pipe before lipid extraction again, then pooled into a tube, next using SpeedVac vacuum concentrator under OH mode for complete drying and stored at -80˚C for sparing until lipidomics analyses. Polar lipids were analyzed using a Exion UPLC system coupled with triple quadrupole/ion trap mass spectrometer (6500 Plus Qtrap; SCIEX) as previous description 47. A Phenomenex Luna 3 µm-silica column (internal diameter, 150x2.0 mm) was used for single lipid classes separation of polar lipids through HPLC using mobile phase A (CCl_4_: CH_3_OH: NH_4_OH = 89.5:10:0.5) and mobile phase B(CCl_4_: CH_3_OH: NH_4_OH: H_2_O = 55:39:0.5:5.5) in proper order. Multiple reaction monitoring transitions were set up for comparative analysis of various polar lipids. Individual lipid species were quantified by referencing to spiked internal standards 48. d9-PC32:0 (16:0/16:0), d9-PC18:0p/18:1, d7-PE33:1 (15:0/18:1), d9-PE18:0p/18:1, d31-PS, d7-PA33:1 (15:0/18:1), d7-PG33:1 (15:0/18:1), d7-PI33:1 (15:0/18:1), C17-SL, d5-CL72:8 (18:2)4, Cer-d18:1/15:0-d7, C12:0 Cer-1-P, d9-SM d18:1/18:1, C8-GluCer, C8-GalCer, d3-LacCer d18:1/16:0, Gb3 d18:1/17:0, d7-LPC18:1, d7-LPE18:1, C17-LPI, C17-LPA, C17-LPS, C17-LPG, d17:1 Sph, d17:1 S1P, GM3-d18:1/18:0-d3 were obtained from Avanti Polar Lipids and Matreya LLC. Free fatty acids were quantitated by d31-16:0 (Sigma-Aldrich; Merck KGaA) and d8-20:4 (Cayman Chemical Company). Free cholesterols and cholesteryl esters were analyzed under atmospheric pressure in chemical ionization mode on a Jasper HPLC coupled to a Sciex 4500 MD 49 and internal standards were d6-cholesterol and d6-C18:0 cholesteryl este51r (CDN isotopes).

### Dual-luciferase Assays

The PANC-1 cells were cultured to 70~80% density and transfected with 0.6 µg constructed luciferase plasmid and *Renilla* luciferase plasmid pRL-TK at a rate of one in ten using Lipofectamine^®^ 3000 reagent. Luciferase activities were detected (Promega Corporation) 40 h after transfection. pGL3-basic empty vector was posed as negative control.

### Electrophoretic mobility shift assay (EMSA)

EMSA was performed as former literature 50. Briefly, single strand primers with biotin labeled at 5' end were dissolved in 1X STE buffer after synthesized (Sangon Biotech Co., Ltd.). Following that, the same amounts of complementary primers were mixed and incubated at 95˚C for 3 min, and gradually recovered to room temperature to generate double-stranded primers (Table S1). Cell nuclear extracts were prepared by nuclear extraction reagents (Beyotime Institute of Biotechnology). EMSA mixture was placed on ice for 10 min by adding or not adding a 200-fold molar excess of unlabeled competitors, then 5'-biotin-labeled double stranded primers were added and the samples incubated for 20 min on ice. For the supershift or immune-depletion assay, 2 μg Sp1 (cat. no. sc-17924X) or Sp3 antibodies (cat. no. sc-28305X) (both from Santa Cruz Biotechnology, Inc) were incubated with the nuclear extracts containing 5 μg protein for 0.5 h at 4 ˚C before adding 5'-biotin-labeled double stranded primers. The mixture was separated by 5% non-denaturing PAGE in 0.5x TAE buffer at 100V at 4˚C for ~90 min, then transferred onto nylon membranes (cat. No. 11209299001; Roche Diagnostics) by semi-dry transfer apparatus (Bio-Rad) at 15V for ~35 min after electrophoresis. Ultraviolet crosslinking for 10 min at room temperature and fixation at 80˚C for 1 h, then Lightshift chemi-luminescence kit (Thermo Fisher Scientific, Inc.) was used for detection.

### RNA interference

Small interfering RNA targeted to Sp1 or Sp3 (Santa Cruz Biotechnology, Inc.) was used to knockdown the expression levels of Sp1 and Sp3, respectively. Non-specific siRNA served as a control. The PANC-1 cell line was transfected with 25 nm Sp1 or Sp3 siRNA (25 nM) using HiPerfect Transfection Reagent (Qiagen Co., Ltd.), then the cells were collected for protein expression analysis or dual-luciferase assay after 40 h.

### Chromatin immunoprecipitation (ChIP) assays

Chromatin isolated from the PANC-1 cell line was applied to the ChIP assays and was carried out in accordance with the instructions (cat. No. 17-10086; Merck KGaA). Primer pairs (CHP-1/CHP-2) were used to amplify the -439/-193 region of *CPTP* promoter. The primer pairs, NS-1/NS-2 were designed to amplify the +67/+251 region of human *CPTP* and acted as negative control without binding sites for Sp1/Sp3 (Table S1). The cycling conditions are as follows: Initial denaturation for 2 min at 95˚C; 30 cycles at 95˚C for 20 sec, 63˚C for 20 sec, and 72˚C for 30 sec; and final extension at 72˚C for 3 min. SYBR qPCR was also performed using recovered DNA for quantitative analysis.

### Western blot analysis

The cells were washed with DPBS twice and lysed in cell lysis buffer which containing 1 mM PMSF and protease inhibitor. The supernatant was collected after centrifugation at cryogenic. Protein was quantified by a BCA kit (Beyotime Institute of Biotechnology). Equivalent amounts of total protein were denatured and separated using 8-12% SDS-PAGE, then transferred onto 0.45-μM NC membranes (Pall Corporation) via semi-dry transfer apparatus. The membranes were incubated at 5% skimmed milk or 4% BSA (Bomei, Inc.) for phosphorylated proteins, then incubated with primary antibodies overnight at 4˚C and secondary HRP-conjugated antibodies for 4 h, at room temperature. ECL substrate (cat. No. 32209; Thermo Fisher Scientific) was utilized to develop the images in a dark room. The following primary antibodies were used: Anti-CPTP (cat. No. HPA056832) from Atlas Antibodies, anti-Sp1 (cat. No. sc-17924X) and anti-Sp3 (cat. No. sc-28305X) from Santa Cruz Biotechnology, anti-E-cadherin (cat. No. 20874-1-AP), anti-α-E-catenin (cat. No. 12831-1-AP), anti-β-catenin (cat. No. 51067-2-AP), anti-Snail (cat. No. 13099-1-AP), anti-vimentin (cat. No. 10366-1-AP), anti-AKT (cat. No. 60203-2-IG), anti-phosphorylated (p)-AKT Ser473 (cat. No. 66444-1-AP) from ProteinTech Group (Wuhan, China), anti-p-AKT Thr308 (cat. No. 9275) and anti- KDM5B (JARID1B; cat. No. 3273) from Cell Signaling Technology, anti-PI4KA (cat. No. ab111565; Abcam), anti-SH3BP1 (cat. No. TA811580S) and anti-β-actin (cat. No. TA-09) from Origene Technologies. The following secondary antibodies were used: goat anti-rabbit IgG (cat. No. EM35111-01) and goat anti-mouse IgG (cat. No. EM35110-01) from Emarbio Science and Technology (Beijing, China).

### Statistical analysis

All experiments were independently repeated 3-6 times. SPSS 19.0 software for statistical analysis was utilized. Student's t test for pairwise comparison and one-way ANOVA for multiple comparisons were executed, respectively. P<0.05 was identified as a statistically different between groups.

## Results

### Increased-*CPTP* expression was associated with a poorer prognosis in PC patients

**Figure 1:**
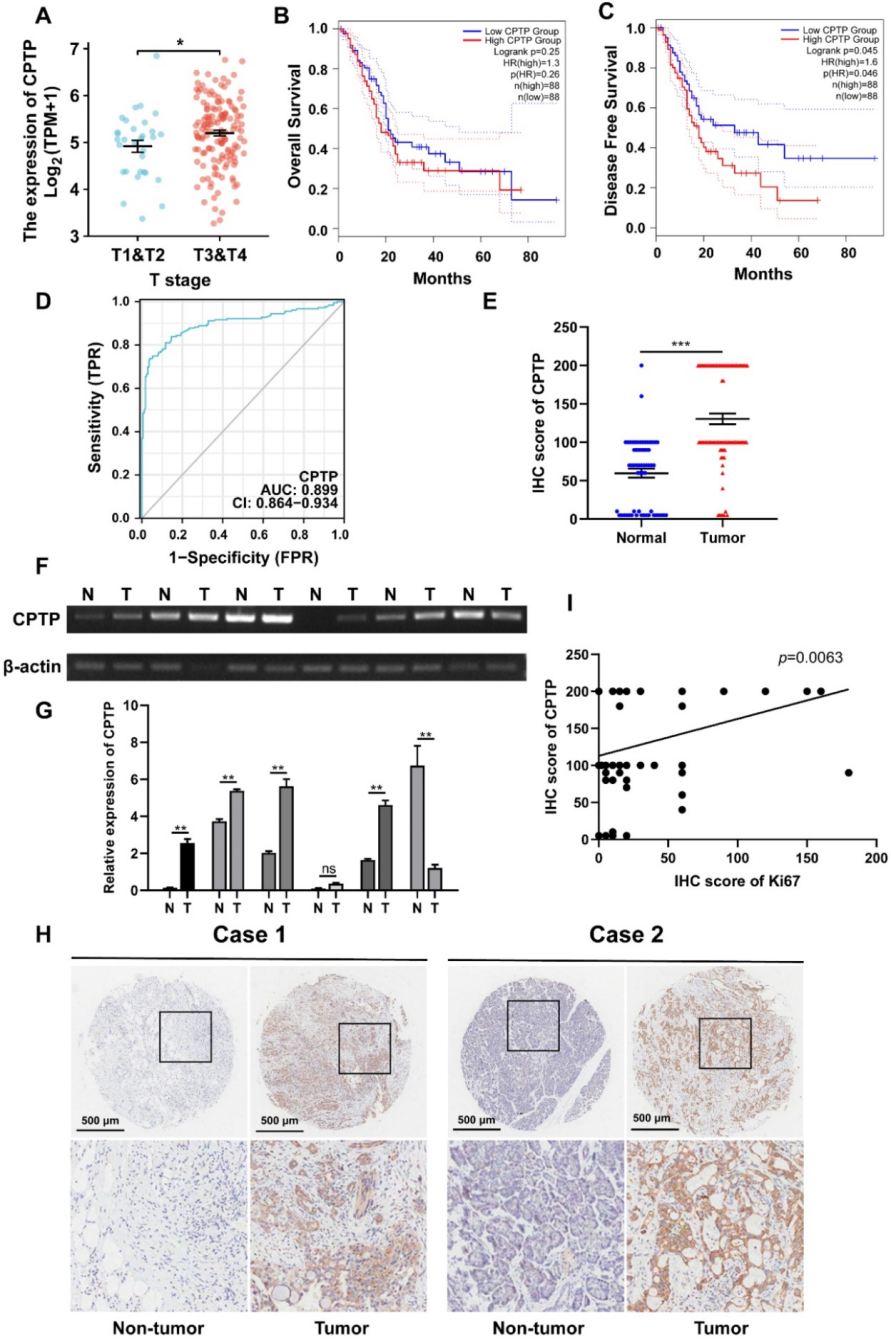
***CPTP* is upregulated in PC tissues and related to poor prognosis of PC patients.** (A) The association between *CPTP* expression levels and tumor T stage from TCGA. The Kaplan-Meier curve was adopted to analyze the association between *CPTP* expression levels and (B) OS and (C) DFS times in patients with PC. (D) Receiver operating characteristic analysis of *CPTP*-based model in predicting clinical outcome. (E) IHC scores of *CPTP* protein expression levels in PC and adjacent normal tissues. (F) Semi-quantitative and (G) quantitative PCR were used to detect the levels of *CPTP* expression in six paired PC tissues and adjacent normal tissues. (H) Representative images of *CPTP* expression in PC and adjacent normal tissues were detected using IHC. Scale bar, 500 μm. (I) Correlation analysis of IHC staining showed a positive correlation between *CPTP* and Ki-67 expression levels. *P < 0.05, **P < 0.01, ***P < 0.001. OS, overall survival; DFS, disease-free survival.

The mRNA expression levels of *CPTP* were increased in PC tissues compared with that in normal tissues according to TCGA 26. To elucidate the association between the *CPTP* mRNA expression level and clinical severity, the *CPTP* expression profile, from TCGA in PC tissues and different stages, was analyzed. *CPTP* was highly expressed in stage T3/T4 samples compared with that in stage T1/T2 samples (Figure 1A). Next, Kaplan-Meier analysis revealed that the high-*CPTP* group had a shorter DFS times compared with the low-CPTP group (*p*=0.046; Figure 1C). There was no significant difference between *CPTP* mRNA expression levels and OS time in patients with PC (Figure 1B). ROC analysis was carried out to evaluate clinical predictive value of *CPTP* in differentiating patients with cancer from those without. As demonstrated in Figure 1D, the area under the curve was 0.899 (95% confidence interval [CI]: 0.864-0.934), and the diagnostic sensitivity and specificity were 73.2% and 96.5%, respectively. This suggested that *CPTP* expression has a high degree of accuracy in tumor prediction. To determine the expression levels of *CPTP* in PC tissues, semi- and qPCR were performed on 6 pairs of PC tissues and the corresponding adjacent normal tissues. The results showed that higher expression levels of *CPTP* were found in four cases of PC tissues. One sample had lower expression levels than the normal (Figure 1F-G). In addition, a TMA, which contained 90 cases of PC tissues and 60 adjacent normal tissues was used to evaluate CPTP protein expression level using IHC. Consistent with the results from TCGA, CPTP protein expression levels were higher in tumor tissues than normal (Figure 1E and H). Furthermore, CPTP was positively associated with the proliferation marker, Ki-67 (Figure 1I). To further confirm the association between CPTP expression and clinicopathological features, the samples were divided into high and low CPTP expression groups, based on the IHC score. As shown in Table 1, the expression levels of CPTP were associated with TNM stage and N stage (N0 and N1). In summary, upregulated* CPTP* expression was associated with poor prognosis of PC patients.

### Overexpression of *CPTP* promotes growth and metastasis of PC cells

**Figure 2:**
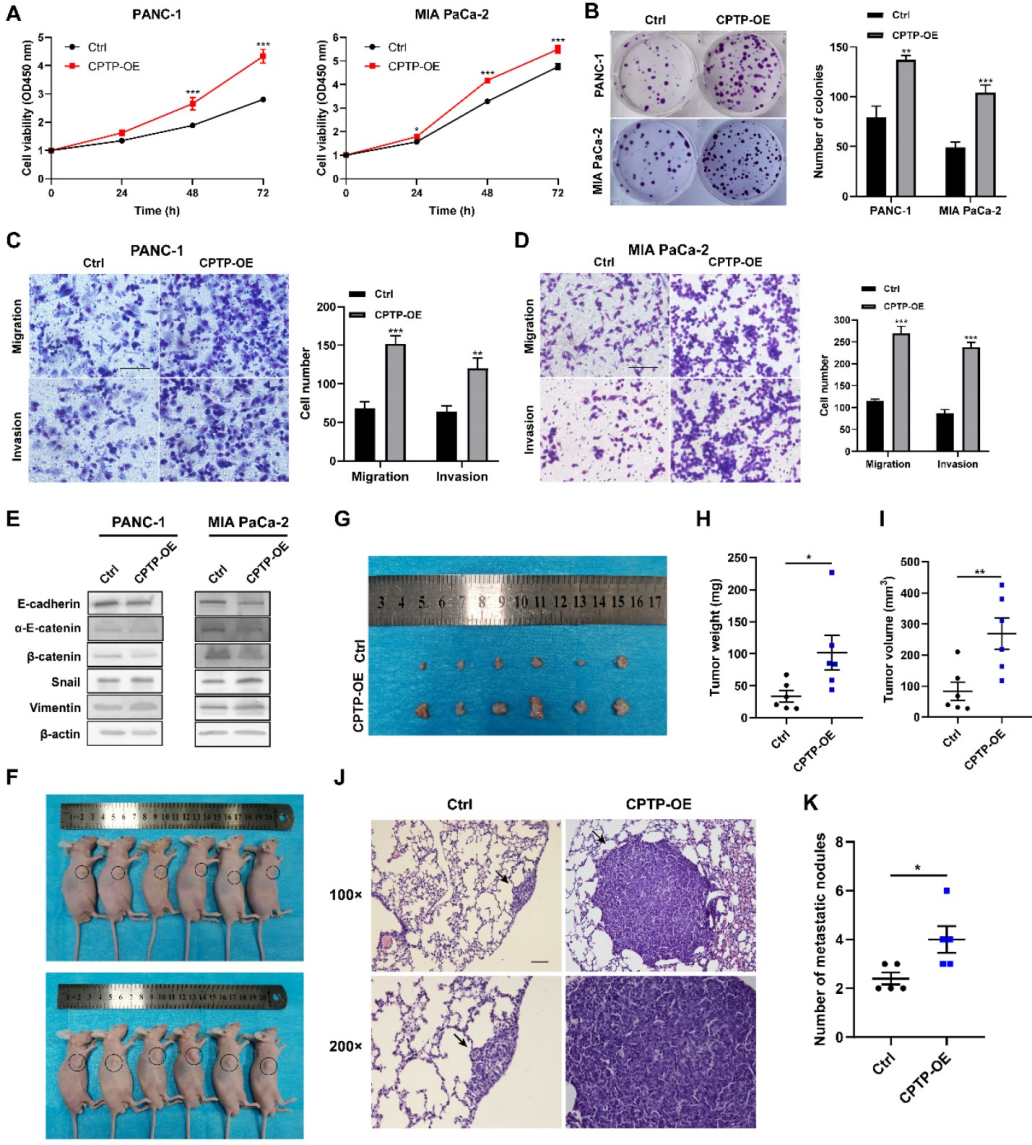
***CPTP* promotes growth and metastasis in PC cells.** The effect of *CPTP* on PC (A) cell proliferation and (B) colony formation ability was determined using Cell Counting Kit-8 and colony formation assays, respectively. The effect of *CPTP* on migration and invasion were evaluated using Transwell and Matrigel assays in the (C) PANC-1 and (D) MIA PaCa-2 cell lines transfected with* CPTP* overexpression plasmid. (E) The relative protein expression levels of E-cadherin, α-E-catenin, β-catenin, Snail and vimentin were analyzed using Western blot analysis following *CPTP* overexpression. (F and G) Images of BALB/c-nude mice in each group 4 weeks after subcutaneous injection of *CPTP* overexpression or control vector in the PANC-1 cells. The tumor (H) weight and (I) volume from mice injected with *CPTP* overexpression vector cells compared with that in mice with empty vector cells. The PANC-1 cells stably overexpressing *CPTP* or the control vector (both 2x10^6^) were subcutaneously injected into the right flank of nude mice. The tumor volume was calculated using the following equation: Volume = (length x width^2^)/2. The mice were injected with *CPTP*-overexpressing PANC-1 cells (5 × 10^6^/mouse) via tail vein to generate a metastasis model, representing H&E staining images of the lung tissues (J) and the number of metastatic nodules (K) in nude mice (n = 5 mice per group, scale bar = 100 μm). The lung metastatic nodules are indicated by the black arrows. *P < 0.05, **P < 0.01, ***P < 0.001. Ctrl, empty vector control; OE, overexpression.

To evaluate the malignant biological behavior of *CPTP* in PC cells, two stable cell lines (PANC-1 and MIA PaCa-2) of *CPTP* overexpressing were constructed by transfecting with pFLAG-CMV4-*CPTP* plasmid (*CPTP*-OE). The expression levels of CPTP in these cells were verified by Western blot analysis (Fig. S1). CCK-8 and colony formation assays showed that *CPTP* significantly promoted PC cells proliferation and colony formation ability compared with the control group (Figure 2A-B). To examine whether *CPTP* might modulate the migration and invasion of the PC cells, Transwell and Matrigel assays were performed. The results demonstrated that *CPTP* overexpression notably increased PC cell migration and invasion (Figure 2C-D). Furthermore, the protein expression levels of epithelial (E-cadherin, α-E-catenin and β-catenin) and mesenchymal (vimentin and snail) cell molecular markers were analyzed to support the aforementioned results. As shown in Figure 2E, the protein expressions of epithelial-related markers were decreased, whilst those of the mesenchymal-related markers were increased. Subsequently, a subcutaneous xenograft assay was performed using nude mice and PANC-1 cells overexpressing *CPTP* to validate the role of *CPTP* in tumorigenesis *in vivo* (Figure 2F-I). After 28 days, the mice with overexpression of *CPTP* had a higher mean tumor weight (101.82±59.99 mg versus the control group 33.4±19.58 mg; *p*=0.0152) and volume (269.25±111.93 mm^3^ versus 83.87±65.36 mm^3^; *p*=0.0095). The maximum tumor volumes and weight were 425.353 mm^3^ and 226.8 mg respectively. Furthermore, CPTP-overexpressing PANC-1 cells had a much higher incidence of lung metastases than control cells (Figure 2J-K). These results indicated that overexpression of *CPTP* promotes growth and metastasis of PC cells.

### Knockdown of *CPTP* suppresses growth and metastasis of PC cells

**Figure 3:**
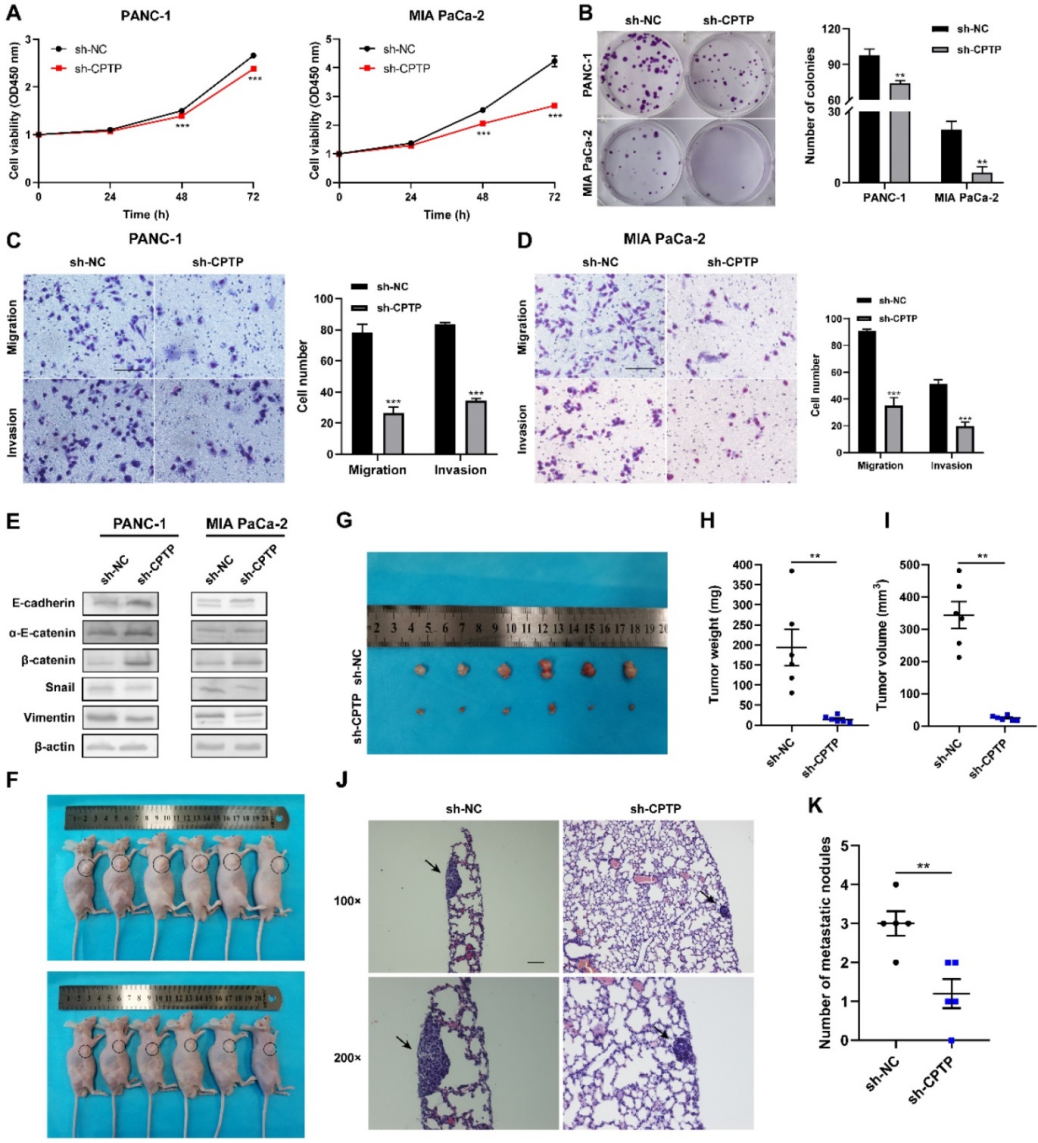
***CPTP* knockdown suppresses growth and metastasis in PC cells.** Cell proliferation and colony formation ability was assessed using a (A) Cell Counting Kit-8 assay and (B) colony formation assays in PC cells transfected with sh-*CPTP* or vector control (sh-NC). (C and D) Cell migration and invasion analysis following *CPTP* knockdown. (E) The relative protein expression levels of E-cadherin, α-E-catenin, β-catenin, Snail and vimentin were analyzed using Western blot analysis in *CPTP* knockdown cell lines. (F and G) Images of BALB/c-nude mice in each group 6 weeks after subcutaneous injection of *CPTP* knockdown or control vector PANC-1 cells. The tumor (H) weight and (I) volume in mice injected with sh-*CPTP* stable cells was compared with that in mice injected with vector control cells. The PANC-1 cells stably expressing sh-*CPTP* and control vector (sh-NC) (both 2x10^6^) were subcutaneously injected into the right flank of BALB/c-nude mice. The tumor volume was calculated using the following equation: Volume = (length x width^2^)/2. For metastasis model establishment, *CPTP*-knockdown PANC-1 cells (5 × 10^6^/mouse) were injected into mice via tail vein (n = 5 mice per group, scale bar = 100 μm), representing pictures of lung tissues by H&E staining (J) and the number of metastatic nodules (K) in nude mice (n = 5 mice per group, scale bar = 100 μm). The lung metastatic nodules are indicated by the black arrows. *P < 0.05, **P < 0.01, ***P < 0.001. NC, negative control; sh, short hairpin.

Stable cell lines (PANC-1-sh-*CPTP* and MIA PaCa-2-sh-*CPTP*), which knocked down the expression levels of *CPTP* by transfecting with shRNAs were established to investigate the effects of CPTP knockdown in the PC cell lines. The expression levels of CPTP in these cells were also determined by Western blot analysis (Fig. S1). CCK-8 and colony formation assays indicated that *CPTP* knockdown markedly inhibited PC cell proliferation and colony formation ability (Figure 3A-B). Furthermore, Transwell and Matrigel assays showed that knockdown of *CPTP* expression suppressed migration and invasion in the PANC-1 and MIA PaCa-2 cell lines (Figure 3C-D). Next, the results from Western blot analyses were consistent with our expectations that knockdown of* CPTP* induced upregulation of epithelial-related markers (E-cadherin, α-E-catenin and β-catenin) and downregulation of mesenchymal-related markers (vimentin and snail) (Figure 3E). In addition, subcutaneous xenograft nude mice experiments revealed that knockdown of *CPTP* inhibited PC tumorigenicity *in vivo* (Figure 3F-G). Tumor weight (14.52±7.84 versus 193.62±100.47 mg) and volume (24.20±6.59 versus 344.12±92.79 mm^3^) was notably decreased in mice transfected with cells and stable knockdown of CPTP expression compared with that in mice transfected with control cells (Figure 3H-I). The maximum tumor volume and weight were 481.263 mm^3^ and 384.5 mg respectively. In addition, the CPTP-knockdown PANC-1 cell group had a considerably lower incidence of lung metastases than the control group (Figure 3J-K). Collectively, these results showed that knockdown of *CPTP* expression suppress growth and metastasis in PC cells*.*

### The effects of *CPTP* overexpression or knockdown on sphingolipid metabolites in PANC-1 cells

**Figure 4:**
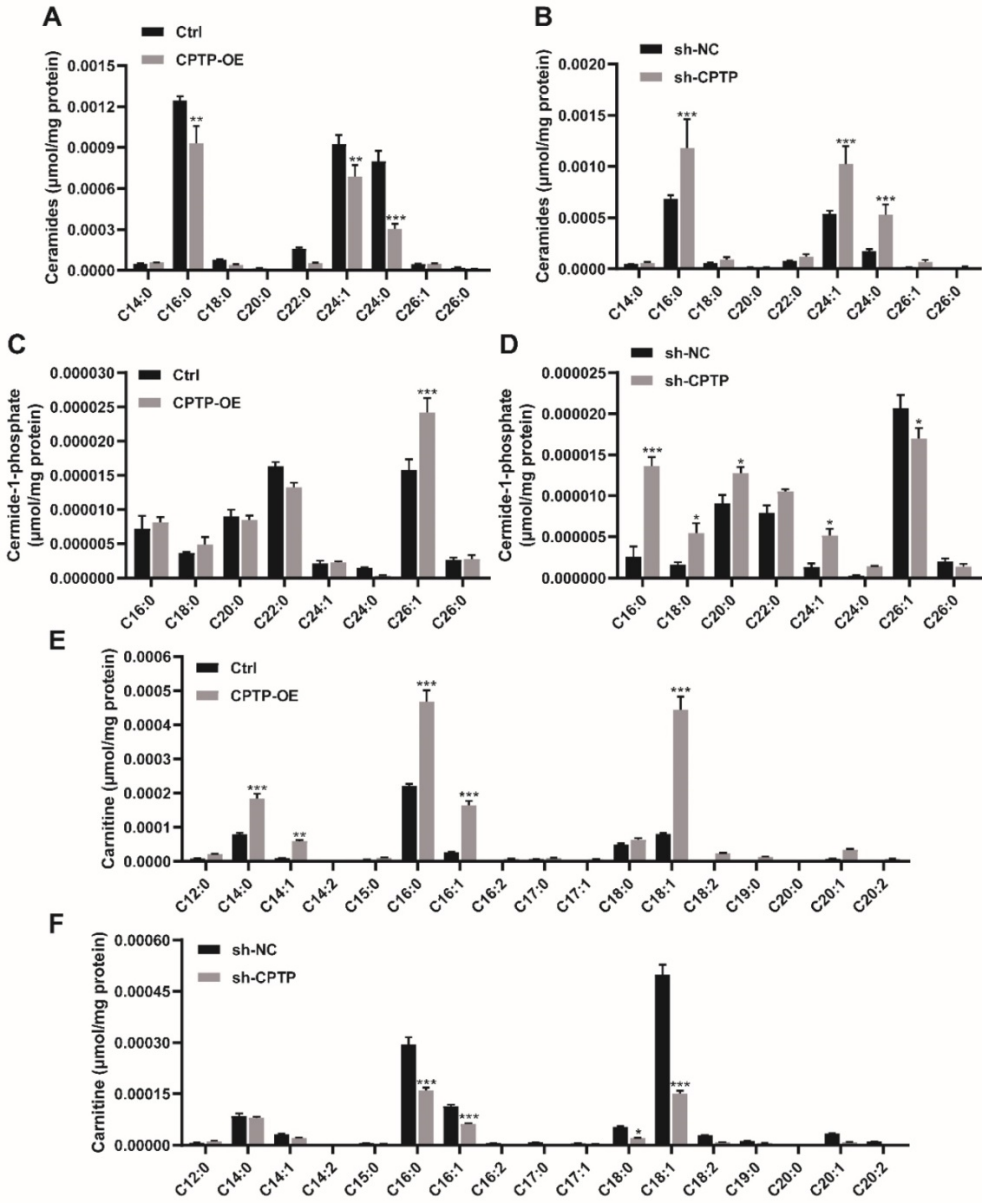
** Effects of CPTP-OE or sh-*CPTP* on sphingolipid metabolites in PANC-1 cells.** Intracellular sphingolipid levels are expressed as micromoles per mg of total protein extracted from the PC cells. (A and B) Ceramide, (C and D) C1P and (E and F) Carnitine levels. The x-axis represents the acyl composition ('C' represents the number of carbon atoms in the fatty acid chain) linked to the sphingosine base chain (d18:1). *P < 0.05, **P < 0.01, ***P < 0.001. Ctrl, empty vector control; OE, overexpression; sh, short hairpin; NC, negative control.

To assess the effect of sphingolipids induced by the overexpression or knockdown of *CPTP* in the PC cells, lipidomics analysis was performed. As shown in Figure 4A, *CPTP* overexpression decreased ceramide levels (16:0-Cer, 24:1-Cer and 24:0-Cer), while *CPTP* knockdown led to an increase in ceramide levels (Figure 4B). As *CPTP* is the only identified protein to transfer C1P, the level of C1P is equally important. Figure 4C illustrates the significant increase of 26:1-C1P in cells transfected with *CPTP* overexpression plasmid compared with the control group, while the opposite results were observed following knockdown of *CPTP* expression. The level of 16:0/18:0/20:0/24:1-C1P was increased in *CPTP*-knockdown cells (Figure 4D). The changes of sphingomyelin (SM), which is one of the main sources of ceramides in the sphingomyelinase pathway (SMase) were also detected. *CPTP* overexpression increased the level of 16:0-SM, 24:1-SM (Figure S2A), the underlying mechanism may be 26:1-C1P elevation inhibited SMase resulting in SM accumulation. In addition, the level of sphingosine (Sph), which is a precursor of S1P, was also measured. C18:1-Sph level was increased in cells overexpressing *CPTP,* whereas the levels were decreased in cells *CPTP* expression knocked down (Figure S2C-D). Notably, the levels of carnitine (14:0/14:1/16:0/16:1/18:1-carniting) and pro-tumor molecular lyso-phosphatidylcholines (C16:1/C18:1-LPC) were increased in cells expressing *CPTP* and decreased in cells with *CPTP* expression knocked down (Figure 4E-F; Figure S2E-F). Carnitine is a cofactor and plays a significant role in fatty acid transfer to mitochondria. Cancer cells utilize fatty acids as an energy source to generate ATP via β-oxidation 51. The aforementioned results indicated that *CPTP* could affect sphingolipid metabolism and is therefore associated with disease progression. In addition, the concentration changes of GluCer, GalCer, LacCer and globoside as well as cholesterol and cholesterol esters were not significant (data not shown).

**Figure 5:**
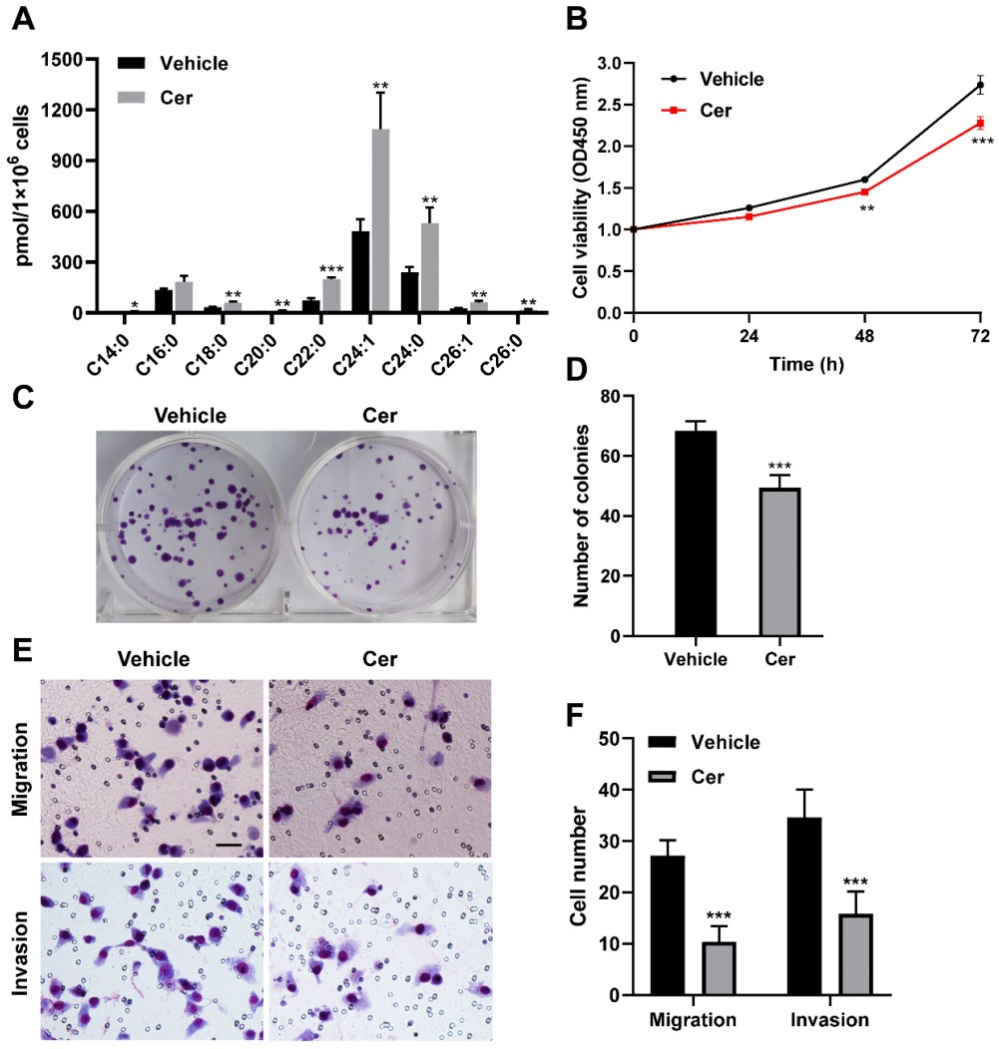
** Increased endogenous ceramide level inhibits cell proliferation, colony formation, migration and invasion in *CPTP* knockdown PANC-1 cells.** *CPTP* knockdown PC cells (PANC-1) were grown to a confluency of 40-60% before being refilled with fresh media. To attain a final concentration of 5 μM, C_6_-ceramide was added to the medium. Cells were collected after lipid treatment for 24 hours and intracellular ceramide levels were measured (A) using HPLC-mass spectrometry, as described in Materials and Methods. Ceramide level is expressed as pmol every 1×10^6^ cells. In the meantime, CCK-8, colony formation, and Transwell assays were used to examine cell proliferation (B), colony formation ability (C-D), cell migration and invasion (E-F) in cells treated with C_6_-ceramide. Cer, C_6_-ceramide. Vehicle, 0.1% DMSO. *P < 0.05, **P < 0.01, ***P < 0.001.

To further investigate the functional implications of SLs concentration changes induced by *CPTP* in PC cells, C_6_-ceramide and carnitine treatment were performed using *CPTP* knockdown PANC-1 cells. C_6_-ceramide treatment resulted in a rise in endogenous ceramide levels, as expected (Figure 5A). Compared with vehicle (DMSO) treatment, more inhibitory effect of C_6_-ceramide treatment was noticed for cells proliferation (Figure 5B), colony formation ability (Figure 5C-D), migration and invasion (Figure 5E-F) in *CPTP* knockdown PANC-1 cells. Nevertheless, the effect of carnitine treatment was not significant for cell proliferation, colony formation ability, migration and invasion (data not shown).

### The effects of *CPTP* overexpression or knockdown on the SH3BP1, KDM5B and PI4KA/AKT signaling pathways in PANC-1 cells

**Figure 6:**
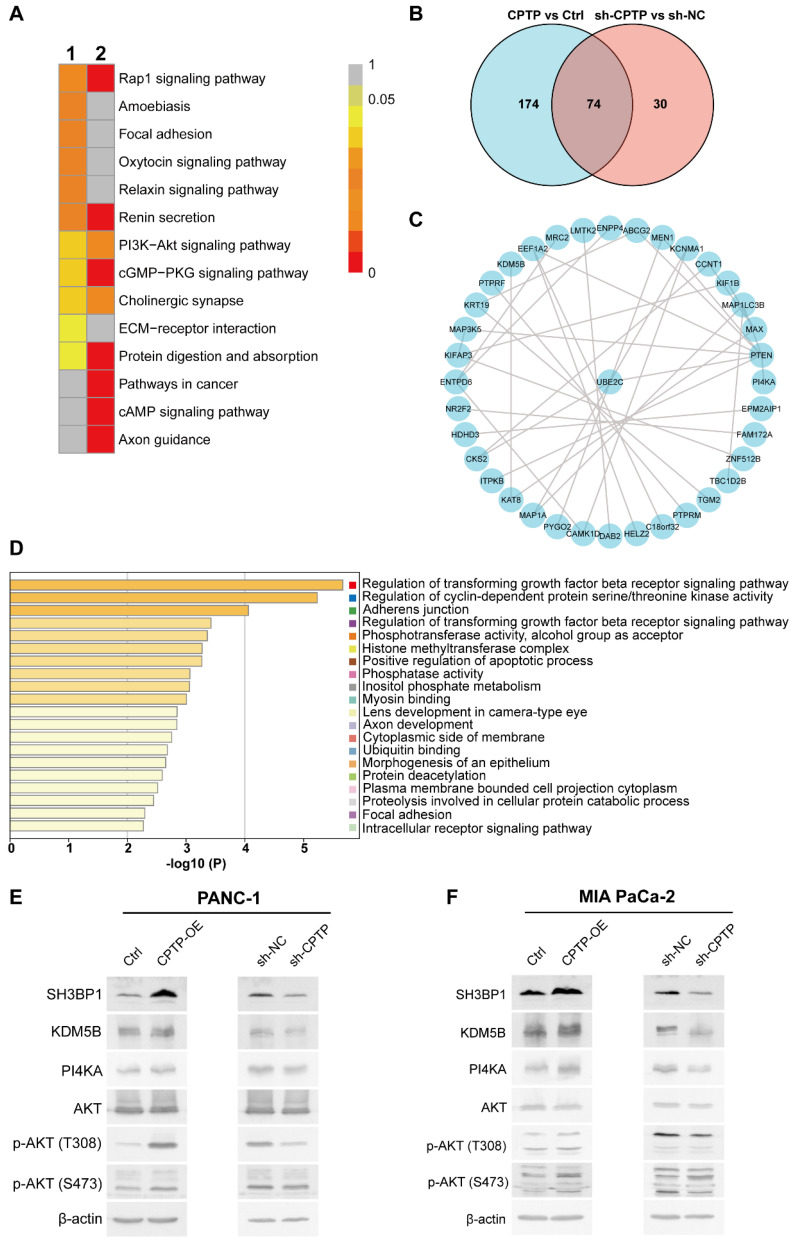
***CPTP* promotes PC cells growth metastasis and may be associated with activation of the serine/threonine kinase or PI4KA/AKT signaling pathways.** (A) The PANC-1 cells stably expressing pFLAG, pFlag-*CPTP*, sh-NC and sh-*CPTP* were analyzed using RNA-Sequencing analysis. KEGG enrichment analysis in cells overexpressing *CPTP* or *CPTP* knockdown was compared with that in the control group in the PANC-1 cell line. (B) Venn diagram showing overlapping genes (n=74) between the differentially expressed genes (at the protein level) from proteome analysis using mass spectrometry in PANC-1 cells transfected with *CPTP* overexpression or knockdown vector compared with that in cells transfected with control vector. (C) Protein-protein interaction of the overlapping genes was analyzed using the Search Tool for the Retrieval of Interacting Genes/proteins database. (D) The enrichment analysis of the overlapping genes was analyzed using GO and KEGG analysis using the Metascape database. The 20 top enrichment terms are shown. (E and F) Experimental validation of serine/threonine kinase activity and PI4KA-AKT signaling pathways using Western blot analysis. Ctrl, empty vector control; OE, overexpression; sh, short hairpin; NC, negative control.

To investigate the molecular mechanism of *CPTP* in promoting PC cells proliferation, migration and invasion, transcriptome and proteomic analysis in the PANC-1 cells with *CPTP* overexpression (*CPTP*-OE) or interference (sh-*CPTP*) was performed. The volcano plots of the differentially expressed transcriptomes are presented in Figure S3A-B. KEGG and GO analysis identified numerous biological processes and signaling pathways that are significantly associated with changes in *CPTP* expression. As shown in Figure S3C-D, *CPTP* was associated with cell migration regulation, which is consistent with the aforementioned results. Subsequently, we found that the PI3K/AKT signaling axis was enriched in the KEGG analysis, with *CPTP* overexpression or knockdown (Figure 6A). The PI3K/AKT signaling axis is often abnormally activated in tumors, suggesting that this pathway may be important in the mechanism of action of *CPTP* in PC. The result prompted us to focus on the PI3K/AKT signaling axis in the subsequent proteome analysis using MS. The top DEPs following *CPTP* overexpression and knockdown are displayed in Tables S2- Table S4, respectively. The Venn diagram showed that 74 differentially expressed genes were obtained from the cells overexpressing *CPTP* or knockdown of expression (Figure 6B). Next, an interaction network of these proteins was produced via the STRING database (Figure 6C). Combined with pathway analysis using the Metascape database (Figure 6D) and the abundance ratio of the differentially expressed genes, the regulation of cyclin-dependent protein serine/threonine kinase activity and inositol phosphate metabolism was selected for Western blot validation. The expression levels of SH3BP1, KDM5B (involved in serine/threonine kinase activity pathway) and PI4KA (belongs to inositol phosphate metabolism pathway) were notably increased in cells overexpressing *CPTP,* and are associated with tumor progression. In addition, high PI4KA expression could activate the PI3K/AKT signaling pathway and induce upregulation of AKT phosphorylation. As expected, the expression levels of SH3BP1, KDM5B and PI4KA were increased in *CPTP*-overexpressing cells, while the protein expression levels were decreased in knockdown cells compared with the control group (Figure 6E-F). The stimulation of pAKT (p-AKT-S473 and p-AKT-T308) was accompanied by high protein expression levels of PI4KA, while decreased p-AKT was observed in cells with *CPTP* expression knocked down. These findings show that *CPTP* overexpression activates the SH3BP1, KDM5B, and PI4KA/AKT signaling pathways in PC cells, but *CPTP* knockdown has the opposite impact. In *CPTP*-overexpressing PANC-1 cells, however, treatment with GSK-A1, a selective inhibitor of PI4KA, decreased AKT phosphorylation, cell proliferation, colony formation, cell migration, and invasion (Figure S4).

### Transcriptional regulation of CPTP expression by Sp1/Sp3 in the PC cells

Firstly, RLM-RACE was performed to characterize the TSS of human CPTP. The reverse transcription reaction was performed utilizing total RNA separated from PANC-1 cells. The first PCR amplification was performed with an outer primer and the RA-1 primer (located in exon 2 of *CPTP*), then the products were used as a template for the second PCR amplification using an inner primer and the RA-2 primer (located in exon 1 of *CPTP*) (Figure S5A). An amplified band of ~140 bp was generated and ligated to T vector, then verified by sequencing. DNA sequencing analysis of 10 randomly selected clones showed transcription start sites of *CPTP* were located at 141 bp (TSS1, 7 clones), 138 bp (TSS2, 2 clones) and 137 bp (TSS3, 1 clone) from the translation start site, ATG of *CPTP* (Figure S5B-C). The results indicated that *CPTP* could be transcribed from at least three start sites and TSS1 stands for the main transcriptional start site.

**Figure 7:**
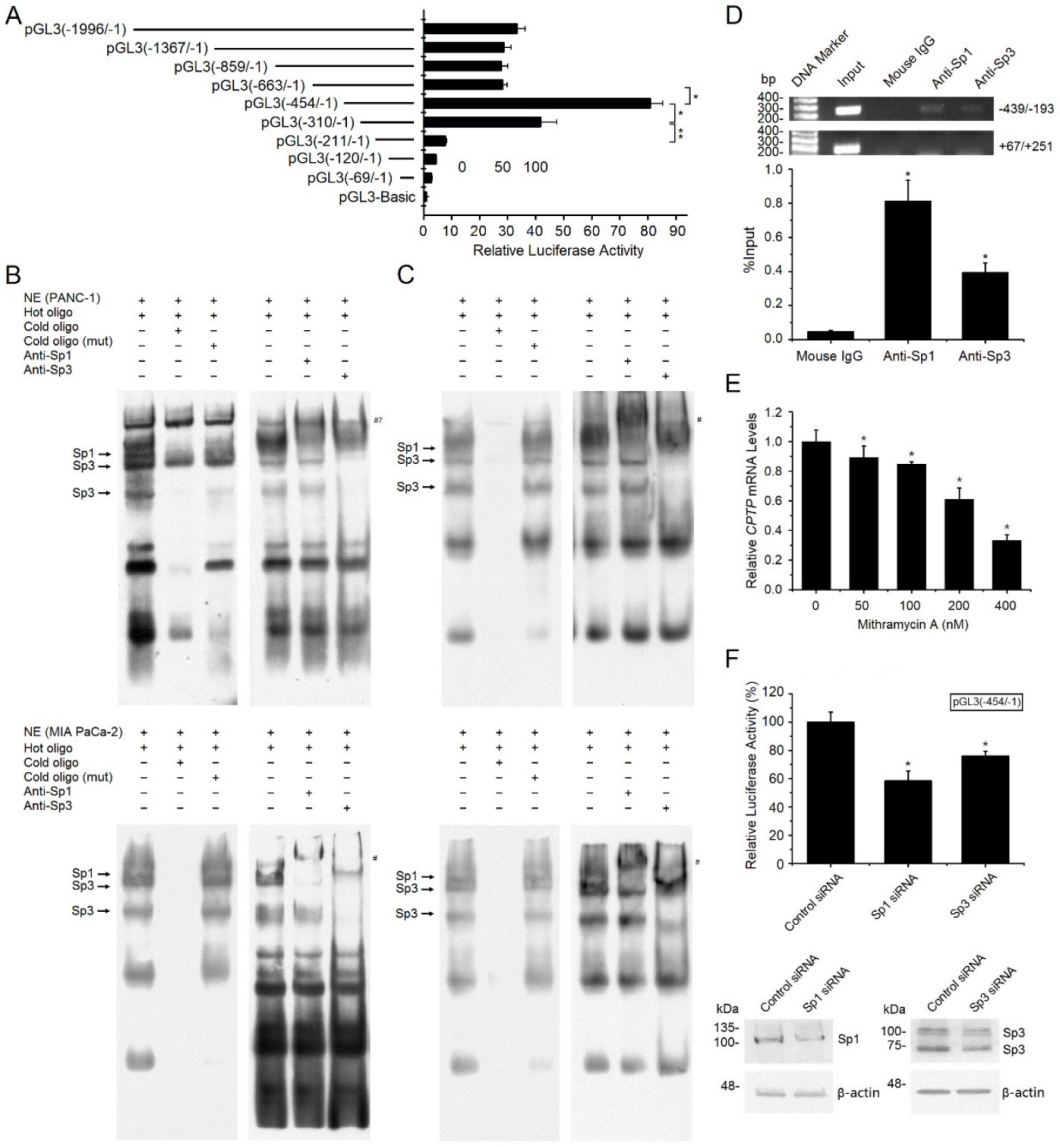
** Transcriptional regulation of *CPTP* expression by Sp1/Sp3.** (A) 5' deletion analysis of the *CPTP* promoter in the PANC-1 cell line. A series of *CPTP* promoter deletion mutants were generated utilizing firefly luciferase plasmid. Each construct was co-transfected with pRL-TK plasmid as the internal control. Numbering is relative to the major transcriptional start site. (B) EMSA for the -282/-273 binding site. The assays were performed with nuclear extracts from the PANC-1 or Miacapa-2 cell lines. Competitive assays were executed with a 200-fold molar excess of unlabeled double-strand probes. Supershift assay was carried out with adding Sp1 or Sp3 antibody. The arrows show specific transcription factor binding. # indicates supershifted bands. #? indicates the position of expected supershifted Sp1 complex. (C) EMSA for the -258/-249 binding site. (D) Sp1/Sp3 interacts with the *CPTP* promoter *in vivo* was identified by ChIP assay, mouse IgG and the *CPTP* region (+67/+251) which does not contain binding sites of Sp1/Sp3 acted as negative control. Input, sheared DNA preceding immunoprecipitation posed as positive control. (E) RT-qPCR analysis for *CPTP* mRNA expression levels in the PANC-1 cells treated with mithramycin A for 24 h. (F) Knockdown of Sp1 or Sp3 decreased *CPTP* promoter activity. Cells transfected with pGL3(-454/-1), then transfected with Sp1 or Sp3 siRNA, and cultured for 24 h before dual-luciferase activity assay and Western blot analysis. *P < 0.05, **P < 0.01.

To identify the proximal regulatory regions of the *CPTP* promoter, 5' flanking region upstream from TSS1 was cloned, and a series of 5' deletion fragments of the putative (-1996/-1) promoter region were constructed. These reporter constructs were transfected into the PANC-1 cells, and luciferase activity was measured after 40 h. Compared with pGL3(-1996/-1), the alteration of promoter activity of pGL3(-1367/-1), pGL3(-859/-1) and pGL3 (-663/-1) was not significant. Deleted regions from -663 to -454 resulted in 2- to 4-fold increase of transcriptional activity compared with that in pGL3(-663/-1) (Figure 7A). This suggests a negative regulatory region in -663/-454 (Figure 7A). Sequence deletion of -454/-211 induced continuous decline of promoter activity. Compared with that in pGL3(-454/-1), the promoter activity of pGL3(-310/-1) was decreased by ~50% (Figure 7A). For pGL3(-211/-1), the promoter activity was also decreased by ~90% (Figure 7A). In conclusion, these results illustrated that the 244 bp region (-454/-211) is the *CPTP* proximal and basal promoter.

Bioinformatics analysis indicated that the -454/-211 region contained two putative Sp1/Sp3 binding sites (-282/-273 and -258/-249). To verify these two Sp1/Sp3 binding sites, nuclear extracts from the PANC-1 and MIA PaCa-2 cells were analyzed using EMSA. Several distinct bands existed (Figure 7B-C). The addition of a 200-fold molar excess of unlabeled probe led to a significant decrease in the bands, while there was no change following the addition of a 200-fold molar excess of unlabeled mutated probe, revealing that these bands are specific binding sites. Generally, the lower mobility of a supershifted Sp1 complex was observed by adding Sp1 antibody, while the original Sp1 complex decreased significantly (Figure 7B-C). Besides, the major Sp3 complex is weakened and the lower Sp3 complex is vanished by adding Sp3 antibody, indicating immunodepletion of Sp1/Sp3 (Figure 7B-C). Collectively, the EMSA analyses indicate that Sp1 and Sp3 bind to these two sites on the CPTP promoter *in vitro* in the PC cells.

ChIP assay was performed to verify the binding *in vivo*. *CPTP* promoter could be detected in the immunoprecipitated complexes by adding Sp1 or Sp3 antibody, but not by adding mouse IgG (Figure 7D). No positive bands appear utilizing a control primer pair specific for CPTP +67/+251 (Figure 7D). The results indicated that Sp1/Sp3 could bind to the proximal promoter of *CPTP in vivo*. Mithramycin-A, an inhibitor of Sp1 binding DNA, reduced *CPTP* mRNA expression levels in a dose-dependent manner (Figure 7E). To evaluate the impact of Sp1/Sp3 on *CPTP* promoter activity, Sp1 or Sp3 siRNA was utilized to knock down gene expression, a 25-50% reduction in *CPTP* promoter activity was observed following knockdown of Sp1/Sp3 expression (Figure 7F). Taken together, these data indicate transcriptional regulation of *CPTP* by Sp1/Sp3 in PC cells.

### Sp3 partially reverses the reduction in cell proliferation, migration and invasion by *CPTP* knockdown

**Figure 8:**
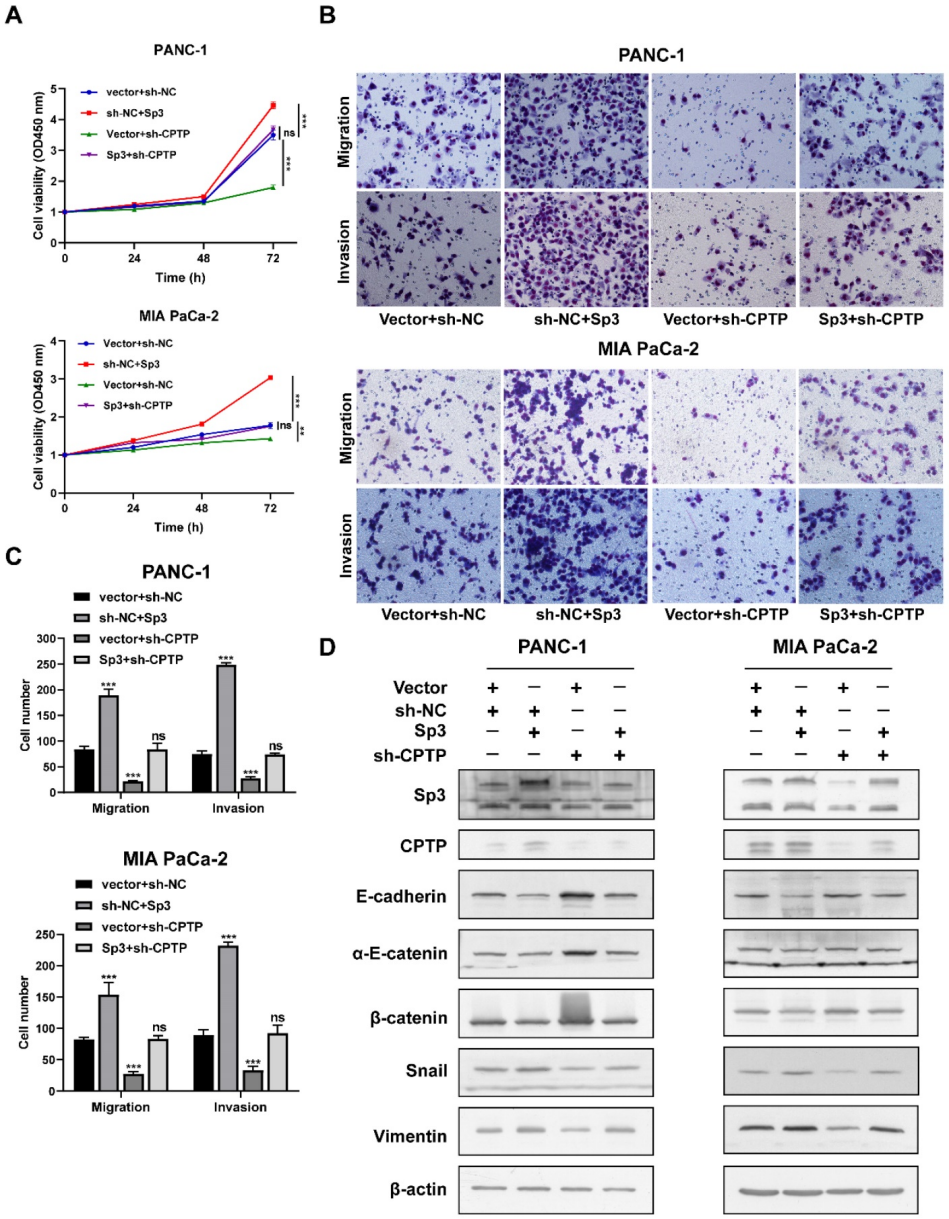
** Sp3 attenuates the decrease in cell proliferation, migration and invasion induced by *CPTP* knockdown.** (A) Decreased cell viability in *CPTP* knockdown PC cells was restored by Sp3 overexpression. The cell viability was measured using a CCK-8 assay. (B-C) Sp3 overexpression rescued the decrease in cell motility in *CPTP* knockdown PC cells. (D) Sp3 overexpression rescued upregulation of epithelial-related markers and downregulation of mesenchymal-related markers expression induced by *CPTP* knockdown as detected by Western blot analysis. *P < 0.05, **P < 0.01, ***P < 0.001. ns, not significant.

As mentioned before, Sp3 is involved in *CPTP* transcriptional regulation. To validate whether Sp3 is involved in the effects of *CPTP* during PC cells proliferation, migration and invasion, a GV141-SP3 plasmid was transfected into *CPTP*-knockdown cells. Sp3 overexpression led to an increase in PC cells proliferation, migration and invasion, and overexpression of Sp3 partially rescued the inhibition of cell proliferation (Figure 8A), migration and invasion (Figure 8B-C) induced following knockdown of *CPTP* expression. Overexpression of Sp3 reversed the *CPTP* knockdown-induced upregulation of epithelial-related markers and downregulation of mesenchymal-related markers (Figure 8D). However, the results from rescue experiments for transcription factor, Sp1 were not statistically significant (data not shown). These results indicated that *CPTP* mediates PC tumorigenesis and is regulated by Sp3.

### Expression levels of Sp1/Sp3 in PC and adjacent normal tissues

**Figure 9:**
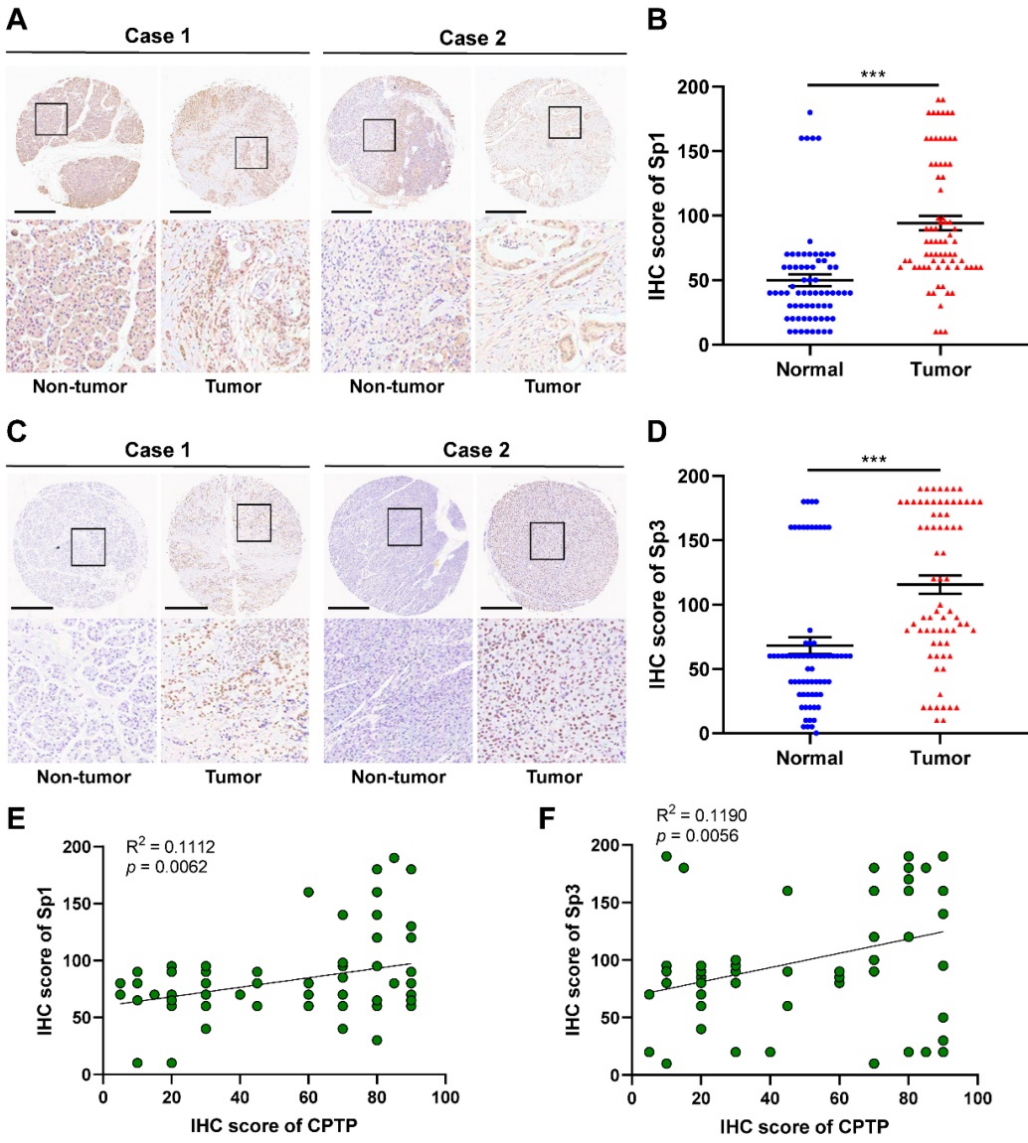
** Sp1/Sp3 expression is concurrently increased with CPTP expression in PC tissues.** Representative images and IHC scores of Sp1 (A-B) or Sp3 (C-D) expression in PC (90 cases) and adjacent normal tissues (60 cases) were detected using IHC. Scale bar, 500 μm. Correlation analysis of protein expression in PC tissue samples between CPTP and Sp1 (E) or Sp3 (F). *P < 0.05, **P < 0.01, ***P < 0.001.

IHC analysis, using tissue sections from PC (90 cases) and adjacent normal tissues (60 cases), was performed to confirm Sp1/Sp3 expression was increased along with *CPTP* expression in PC tissues. Compared with that in adjacent normal tissues, Sp1/Sp3 protein expression levels were also significantly higher in tumor tissues (Figure 9A-D). The expression levels of Sp1 are associated with tumor grade and M stage, and the expression levels of Sp3 were relevant to tumor grade and TNM stage (Tables S5 and S6) was observed by the clinicopathological characteristics analysis of PC patients. Meanwhile, in PC tissue samples CPTP expression was closely linked to Sp1 or Sp3 expression (Figure 9E-F). These results indicated that the transcription factors, Sp1 and Sp3 could indirectly promote metastasis by increasing the expression levels of *CPTP* in PC cells.

## Discussion

GLTPs were identified over thirty years ago. The GLTP family members are involved in sphingolipid homeostasis, inflammation, autophagy and necroptosis induction in certain colon cancer cell lines; however, the understanding of their *in vivo* functional roles and clinical application has been comparatively slow 28. CPTP belongs to the GLTP family and is the only currently identified protein to transfer C1P 27. *CPTP* knockdown triggers inflammation and autophagy 36, and *CPTP* has been associated with tumor development and progression 27. Notably, *CPTP* has been considered as a promising molecule of cancer 52; however, the role and molecular mechanism involved remain elusive.

In this research, *CPTP* was first confirmed to act as a pro-tumor molecule and plays an important role in PC. The results showed that *CPTP* expression is increased in PC tissues, although there was one case in which expression was decreased. This could be due to individual differences. CPTP expression was also associated with tumor TNM stage, suggesting that *CPTP* expression is related to dismal prognosis in patients with PC. In addition, the distant metastasis to organs such as liver and lung is common in PDAC, especially in late stages 6. Therefore, it is necessary to clarify the relationship between *CPTP* expression and the distant metastasis in further clinicopathological studies.

*CPTP* knockdown increased intracellular C1P levels (C16:0, C24:1) in the A549 cell line; however, there was no significant change in *CPTP*-overexpressing cells 27. The intracellular C1P levels in the present study are mainly consistent with previous reports 27. However, the level of C26:1-C1P was elevated in *CPTP*-overexpressing cells and decreased in *CPTP* knockdown cells in this study. C1P facilitates cell growth, migration, differentiation and survival via the PI3K/AKT/mTOR, glycogen synthase kinase-3β or Ras/Raf/ MEK/ERK pathways, which are well-known to have tumor-promoting roles in human cancer 26, 29, 30, 53-57. *CPTP* knockdown induced C24:0-ceramide downregulation and C24:1-ceramide upregulation in the A549 cell line 27. However, our results showed that ceramide levels were decreased in *CPTP*-overexpressing cells and increased in *CPTP* knockdown cells. Ceramide can induce apoptosis in various cancer cells and is recognized as a tumor suppresser 58. Mechanistically, Bax is recruited to the mitochondrial outer membrane following the accumulation of ceramide in the mitochondria, leading to mitochondrial outer membrane permeabilization and cell death in a caspase-3-dependent manner 59, 60. Alternatively, AKT inhibits the transfer of Bax to the mitochondria and leads to caspase-9 activation, which is upstream of the caspase-3 cascade 61, 62. This may be a potential mechanism to explain the results in this study. Notably, upregulation of *CPTP* expression led to the increase in carnitine level; however, this phenomenon has not yet been reported for lipid transfer proteins. Meanwhile, significant effect of C_6_-ceramide treatment (Figure 5) 50, and unnoticeable effect for carnitine treatment 63 were observed in this study (data not shown). These results indicated that the CPTP-induced SL concentration changes produced a complex impact on the cellular function. Metabolic flexibility has been previously associated with cancer, due to the high energy required for proliferation and increased malignancy of cancer cells 64. The Warburg effect is known to be the main energy source and a hallmark of cancer progression; however, reprogramming energy metabolism is also an emerging hallmark for tumorigenesis 65. Fatty acid oxidation (FAO) is another main efficient energy-producing method in addition to glycolysis in cells 66. For instance, prostate cancer and diffuse large B-cell lymphoma cells utilize FAO to meet the ATP requirement via upregulation of related action enzymes 67, 68. Carnitine is sufficient and necessary for FAO by the mitochondria, and it carries acyl moieties of fatty acid into the mitochondrial matrix, the place where β-oxidation occurs 51, 69. These results suggested that the elevated carnitine levels caused by *CPTP* overexpression might be important for energy consumption in PC cells.

The results of lipidomic analysis showed that the levels of Sph and LPC were increased in cells following *CPTP* overexpression. Sph could be converted to S1P via sphingosine kinase. S1P regulates multiple biological processes associated with tumor initiation and progression, such as angiogenesis and tumor metastasis 32; however, the association between Sph and cancer remains to be clarified. LPCs are highly expressed in plasma or urine in patients with ovarian cancer compared with that in healthy controls and act as signaling molecules to facilitate cell proliferation, angiogenesis, migration, inflammation and wound healing 70, 71. A recent study showed that LPCs were associated with lower risk of breast, colorectal and prostate cancers, and both endometrioid/clear cell and serous/poorly differentiated ovarian tumors 71. This suggests that LPCs are associated with tumors; however, larger studies are required to assess LPCs as potential biomarkers for cancer.

The expression of C1P with very long acyl chains is affected by the changes in the expression levels of *CPTP*, while SM is affected and increased when *CPTP* is overexpressed. Based on our proteomic analysis data, the expression levels of ceramide synthases and CERT were not significantly affected by the CPTP overexpression (data not shown). The reason for the increased SM, such as *de novo* SM synthesis, or SMase generated SM, remained to be further investigated.

**Figure 10:**
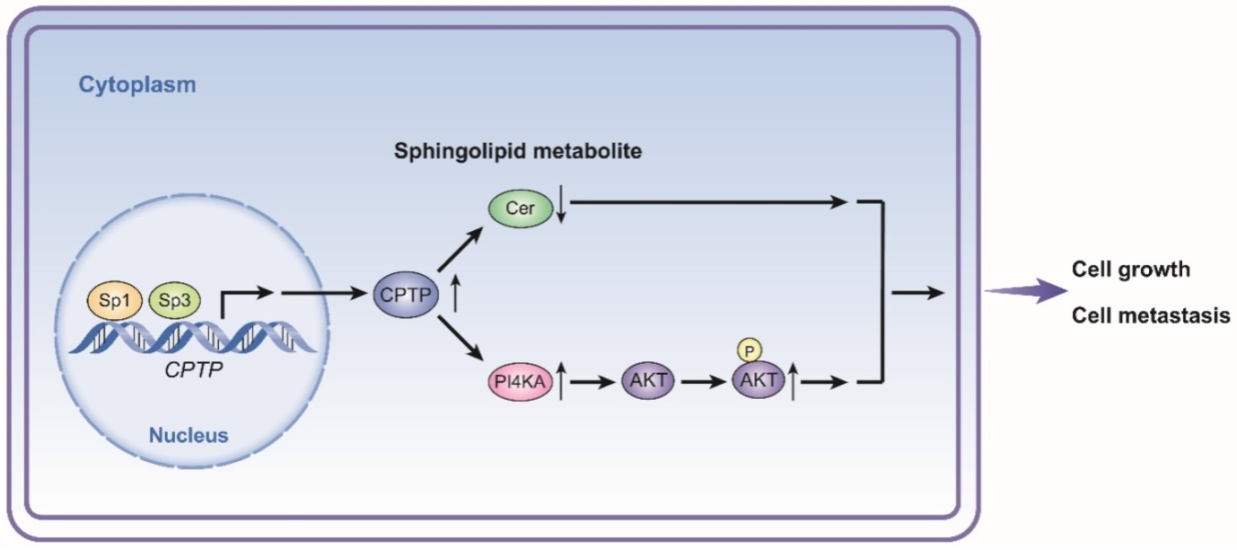
** Schematic diagram illustrating the proposed mechanism of *CPTP* in PC cells.** *CPTP* expression affects the levels of sphingolipid metabolite ceramide as well as the PI4KA/AKT signaling pathways. At the point when *CPTP* expression is upregulated, activation of the PI4KA/AKT signaling pathway and decreased levels of the sphingolipid metabolite ceramide promote PC cell growth and metastasis, respectively. Furthermore, in PC cells, the transcription factors Sp1 and Sp3 operate as upstream positive regulators of *CPTP* expression. Cer, ceramide.

Abnormal activation of SH3BP1 and KDM5B, which are serine/threonine kinase signaling members, and PI4KA/AKT signaling was observed following alteration of *CPTP* expression (Figure 10). SH3BP1 belongs to the Rho GTPase activating protein family and mediates cell motility 72, in a similar manner to that observed during EMT in prostate cancer, hepatocellular carcinoma (HCC) and cervical cancer via the Rac family small GTPase 1-WASP family member 2 pathway 73-75. KDM5B is a histone tri- or di-methylated H3K4 (H3K4me3/2) demethylase, which promotes gastric and prostate tumorigenesis via the hyperactivation of PI3K/AKT signaling 76-78. KDM5B is abundant in PC tissues 79 and causes EMT in PC cells (such as PANC-1 cells) 80. PI4KA is one of the upstream kinases of phosphatidylinositol (PI), (which phosphorylates PI into phosphatidylinositol-4-phosphate at the D4 position); thereby inducing PI3K/AKT signaling pathway activation in PC cells 81. In another study, PI4KA exhibited biological significance in hematopoiesis, and AKT signaling and abnormal expression of PI4KA was associated with hematological malignancies 82. Meanwhile, the laminin subunit of beta-3 gene mediates cell proliferation and migration/invasion in PC cells by activating PI3K/AKT signaling 83. Our results indicate that CPTP regulates cell proliferation, migration/invasion, and AKT phosphorylation in PANC-1 cells through activating PI4KA/AKT signaling, and that blocking PI4KA reduces AKT phosphorylation, cell proliferation, colony formation, cell migration, and invasion. On the other hand, the supplementation of carnitine leads to an activation of the IGF-1/PI3K/AKT signaling pathway using rat animal models 84. Our results of PI4KA mediated PI3K pathway were observed using human PC cells and it might be activated by the alterations of multiple factors induced by *CPTP*. In the meanwhile, more studies are needed to confirm the functions of KDM5B and SHBP3 signaling in PC cells.

To further understand the role of *CPTP* in the initiation and progression of PC, transcriptional regulation of *CPTP* expression in the PC cell lines was investigated. Our results revealed at least three transcriptional start sites; however, the number of known start sites in the Genbank database is higher. The GC rich 5' untranslated region of *CPTP* (75.7% for the CpG island and 93% in some regions) was difficult to amplify (Figure S6), similar to *GLTP* 50. Our results indicated that *CPTP* has no TATA-box in the promoter and the basal promoter contains at least two Sp1/Sp3 binding sites. The results also revealed that Sp1/Sp3 binds to these two sites* in vitro* and *in vivo*, and both Sp1 and Sp3 are transcriptional activators for *CPTP* (Figure 7). With Sp1 immunodepletion, the supershifted bands of Sp1 for the -282/-273 binding site using the PANC-1 cell line were not strong, while the supershifted bands were clear for the MIA PaCa-2 cell line (Figure 7B). This might be due to the overlap of the non-specific band position with the supershifted bands and the Sp1-antibody-probe complexes are not stable in the nuclear extracts from the PANC-1 cell line. Co-regulatory factors, such as sterol regulatory element-binding proteins, which bind to Sp1 to regulate transcription of *CPTP* 85, and competitive transcription factors, such as Krüppel-like factors could complicate the regulatory process 86, 87. It has been shown that Sp1/Sp3 plays important roles in cell proliferation and metastasis in various tumors 88-90. The results indicated that Sp3 partially reverses the decreased cell proliferation, migration and invasion induced by knockdown of *CPTP*, while Sp1 could not significantly reverse cell proliferation, migration and invasion induced by *CPTP* knockdown. This could be due to the regulation of expression of multiple genes by Sp1 and the effects of these genes could offset the effects induced following knockdown of *CPTP* expression. Our results also indicated that Sp1/Sp3 expression is concurrently elevated with *CPTP* in PC tissues compared with the normal (Figure 9). Collectively, these results suggest that Sp1/Sp3 plays a role in the increase of *CPTP* expression during the initiation and progression of PC (Figure 10).

Taken together, the results of this study showed that *CPTP* may function as a pro-tumorigenic gene, and that this process is regulated by transcription factors, Sp1/Sp3 in the PC cells. In PC cells, CPTP promotes growth and metastasis through the sphingolipid metabolite ceramide and PI4KA/AKT signaling. Meanwhile, the promoting effect of *CPTP* in PC development could be the result of multiple effects. The present study provides evidence for *CPTP* as a biomarker and candidate therapeutic target of PC; however, further investigation is required into the suppression of metastasis by targeting *CPTP* and increased patient survival in follow-up studies.

## Supplementary Material

Supplementary methods, figures and tables.

## Figures and Tables

**Table 1:** Correlation between *CPTP* expression and clinicopathological characteristics for PC patients

	variables	*CPTP* expression		total	χ^2^	p value^ a^
		low	high			
Age (year)					0.026	0.871
	≤62	5	39	44		
	>62	4	35	39		
Sex					1.263	0.261
	Female	8	45	53		
	male	3	28	31		
Grade					0.161	0.688
	I/II	7	47	54		
	III/Ⅳ	3	27	30		
T stage					0.162	0.687
	T1/T2	8	62	70		
	T3	2	11	13		
N stage					7.476	0.006**
	N0	1	41	42		
	N1	8	28	36		
TNM stage					4.973	0.026*
	Ι	1	34	35		
	II	9	38	47		
Tumor size					0.135	0.713
	≤4cm	5	41	46		
	>4cm	5	32	37		

* P < 0.05; **P < 0.01 ^a^ Chi-squared test results
