# Supplementary material for: Human CPTP promotes growth and metastasis via sphingolipid metabolite ceramide and PI4KA/AKT signaling in pancreatic cancer cells
Source: Int J Biol Sci. 2022 Jul 27;18(13):4963–83. doi: 10.7150/ijbs.70007 (PMC9379408; doi:10.7150/ijbs.70007)
Supplement: Supplementary file 1 — Supplementary methods, figures and tables. [file ijbsv18p4963s1.pdf]

Human *CPTP* promotes growth and metastasis via sphingolipid metabolite ceramide and PI4KA/AKT signaling in pancreatic cancer cells

Yanqun Zhang[1], Shenying Ji[2,3], Xiangyu Zhang[2,3], Mengyun Lu[2,3], Yihong Hu[2,3], Yucheng Han[2,3], Guanghou Shui[4], Sin Man Lam[5] and Xianqiong Zou[2,3*]

1. Department of Oncology, Xiangya Hospital, Central South University, Changsha 410008, Hunan, P. R. China
2. Affiliated Stomatology Hospital of Guilin Medical University, Guilin 541004, Guangxi, P. R. China
3. School of Basic Medical Sciences, Guilin Medical University, Guilin 541100, Guangxi, P. R. China
4. State Key Laboratory of Molecular Developmental Biology, Institute of Genetics and Develop mental Biology, Chinese Academy of Sciences, Beijing 100101, P. R. China
5. State Key Laboratory of Molecular Developmental Biology, Institute of Genetics and Developmental Biology, Chinese Academy of Sciences, Beijing 100101, P. R. China; LipidALL Technologies Company Limited, Changzhou 213022, Jiangsu, P. R. China

* Corresponding author: Affiliated Stomatology Hospital of Guilin Medical University, Guilin 541004, P. R. China. E-mail: zouxq019@glmc.edu.cn

Material and methods

Plasmids—Open reading frames of human *CPTP* were cloned into pFlag-CMV4 via the HindIII and BamHI restriction sites (Table S1). sh-CPTP for silencing human CPTP was constructed as described previously [27]. Human blood genomic DNA (Promega) was used as template, a 1996 bp PCR fragment (primer pair DP-1/DP-2) was amplified using Advantage GC Genomic LA polymerase mix (Clontech), which was then cloned to pGL3-basic (Promega Corporation) by using the KpnI and HindIII to construct pGL3(-1996/-1). The following amplified conditions were used: Pre-denaturation: 2 min at 94˚C, followed by 38 cycles at 94˚C for 30 sec, 60˚C for 30 sec and 72˚C for 120 sec, and extension at 72˚C for 5 min. 5' deletion mutants of CPTP promoter, fragments of 6,177 bp (D-1/AL1), 5,669 bp (D-2/AL1), 5,473 bp (D-3/AL1), 5,264 bp (D-4/AL1), 5,120 bp (D-5/AL1), 5,021 bp (D-6/AL1), 4,930 bp (D-7/AL1) and 4,879 bp (D-8/AL1) were amplified by Herculase (Agilent Technologies) supplemented with betaine (Sigma) from pGL3(-1996/-1), respectively (Table 1). pGL3(-1367/-1), pGL3(-859/-1), pGL3(-663/-1), pGL3(-454/-1), pGL3(-310/-1), pGL3(-211/-1), pGL3(-120/-1) and pGL3(-69/-1) were produced by self-cyclization, respectively. For amplification of self-cyclization fragments, the cycling conditions were performed: pre-denaturation: 2 min at 94˚C, followed by 36 cycles at 94˚C for 20 sec, 63˚C for 30 sec and 72˚C for 200 sec, final extension at 72˚C for 5 min. All plasmid constructs were confirmed by sequencing (Shanghai Sangon Biotech). GV141-Sp1, GV141-Sp3 and GV141 control plasmid were purchased from Genechem (Shanghai, China).

Rapid amplification of cDNA ends assay (RACE)—Total RNA was extracted from PANC-1 cells using Trizol reagent (Thermo Fisher Scientific). Reference to the manufacturer's suggestions, a RNA ligase mediated-RACE was performed using a FirstChoice RACE kit (Invitrogen). Advantage GC Genomic LA polymerase mix (Clontech, Mountain View, CA, USA) was used for amplification. Primer RA-1 and RA-2 (Table 1) were used for first- and

second-round PCR amplifications. The amplified products were separated on 1.2% agarose gel by electrophoresis, the purified products were cloned into pGEM-T vector (Promega, Madison WI) and plasmids were sequenced (Sangon Biotech Co., Ltd., Shanghai, China).

Lipid effects on cell proliferation, colony formation ability, migration and invasion—CPTP knockdown PC cells (PANC-1) were grown to 40~60% confluence, and then recharged with fresh medium. C_6-ceramide and carnitine (Sigma) were added into the medium reaching a final concentration of 5 μM (DMSO-treated as controls) and 500 μM, respectively. After lipid treatment for 24 h, cells were collected and analyzed for intracellular ceramide levels. Meanwhile, cell proliferation, colony formation ability and migration were assessed using Cell Counting Kit (CCK)-8, colony formation and Transwell assays, respectively.

Bioinformatics analysis—CpG island region of human *CPTP* was analyzed using MethPrimer. GC content contribution of the CpG island was caculated using GC content calculator.

Supplementary Table 1: Primers used in this study.

Name	Oligonucleotide sequence (5'-3')[a]	Position[b]
Primers for pFlag-CPTP construction		
Flag-1	CCCAAGCTTATGGATGACTCGGAGACAGGTTTCA	
Flag-2	CGGGATCCCTAGGGCAGGTCCAGCAGGGAG	
Primers for CPTP-shRNA (pSuper.CPTP.puro-egfp [27] construction		
Primers for CPTP of *RT-qPCR [27]*		
Primers for ACTB control of RT-qPCR [42]		
Primers for 5'-RACE analysis		
RA-1(antisense)	TTGAAACCTGTCTCCGAGTCATCCA	+143/+167
RA-2(antisense)	AGATGGCACCGGACTGGATGGG	+29/+50
Primers for 5'-deletion constructs		
DP-1(sense)	CGGGGTACCACGGAGGACCCAGAGAGCAGGG	-1996/-1975
DP-2(antisense)	CCCAAGCTTAGGCCCAGGAAGGGGCGGAGG	-1/-21
D-1 (sense)	CAAAGTGCTGTGATTGCAGGCGTG	-1367/-1344
D-2 (sense)	GGGAGGCTGAGAGGCTGGGGA	-859/-839
D-3 (sense)	CTCGCGTTCTCGCGTCACTGCC	-663/-642
D-4 (sense)	AAGGGCGTGACTCTGATCTCAGGCA	-454/-430
D-5 (sense)	CTCCTGATTGGGCAGCATCCAACC	-310/-287
D-6 (sense)	TAGGTGAGCGGCTCGGACTCGG	-211/-190
D-7 (sense)	TCGTCCTAGAGGGCCGGAGCG	-120/-100
D-8 (sense)	AGGACGGAGCCGTGGCTCAGGTC	-69/-47
Primer AL1 [50]		
Primers for EMSA assay		
EM1 (sense)	ACCAATCAGGGCGGCGGGCGAG	-289/-268
mEM1 (sense)	ACCAATCAGAAAAAAGGGCGAG	-289/-268
EM2 (sense)	GGGGCGAGGGCGGGGCGGTGG	-264/-244
mEM4 (sense)	GGGGCGAGAAAAAAGCGGTGG	-264/-244
Primers for CHIP assay		
CHP-1 (sense)	ATCTCAGGCATCGTCTCCGCCG	-439/-418
CHP-2 (antisense)	AGTCCGAGCCGCTCACCTAGGC	-193/-214
NS-1 (sense)	AAAATGAGCCACAGAGCAAGCTGACC	+67/+92
NS-2 (sense)	TTCCAGCTGGCAATGTAGGGGTC	+251/+229

[a]Oligonucleotides not derived from human *CPTP* are underlined.

[b]Position is relative to major transcriptional start site of human *CPTP* (=+1).

Supplementary Table 2: The top upregulated proteins in *CPTP*-overexpressing cells.

Gene Symbol	Abundance Ratio	Gene Symbol	Abundance Ratio
RAP1GAP2	100	*LONP2*	100
LMBRD2	100	*KRT2*	100
LMTK2	100	*EPM2AIP1*	100
ZNF512B	100	*CAMK4*	100
PHKG2	100	*KCNMA1*	100
PDZD8	100	*TBC1D2B*	100
INPP5B	100	*NTN4*	100
FMNL1	100	*MRC2*	100
CDC42EP4	100	*PI4KA*	100
SUOX	100	*KAT8*	100
NEXN	100	*CD69*	100
MAX	100	*MAP3K5*	100
ZFX	100	*FLYWCH1*	100
CNPY4	100	*PCNT*	100
KDM5B	100	*DAB2*	100
PTPRM	100	*SH3BP1*	100
LYST	100	*SPRED2*	100
LAT2	100	*FARP1*	100
MAP1A	100	*PTEN*	100
TTC31	100	*CACUL1*	100
FASTKD1	100	*KIF1B*	100
ITPKB	100	*ENTPD6*	100
NR2C2	100	*PDP1*	100
C18orf32	100	*MEN1*	100
RNF139	100	*FAM172A*	100
DOCK10	100	*N4BP2*	100
KRT14	100	*NAGA*	100
RCOR3	100	*UBXN6*	100
TMEM41A	100	*PYGO2*	100
SMARCAD1	100	*SACS*	100
CAMK1D	100	*CASZ1*	100
RAB3IL1	100		

Supplementary Table 3: The top downregulated proteins in *CPTP*-overexpressing cells.

Gene Symbol	Abundance Ratio	Gene Symbol	Abundance Ratio
PLIN2	0.01	*CKS2*	0.01
CCPG1	0.01	*PTX3*	0.01
YIPF5	0.01	*GPNMB*	0.01
CCNT1	0.01	*TTC28*	0.01
CASP4	0.01	*HDHD3*	0.01
RAB2B	0.01	*RBPMS*	0.01
RAB24	0.01	*RRP8*	0.01
SHROOM1	0.01	*VGF*	0.01
RBCK1	0.01	*UVRAG*	0.01
TNFSF9	0.01	*DHDDS*	0.01
HELZ2	0.01	*LRP10*	0.01
ABCG2	0.01	*NBR1*	0.01
NR2F2	0.01	*KRT15*	0.01
TUBGCP4	0.01	*PTPRS*	0.01
ENPP4	0.01	*DNAJC15*	0.01
DTNB	0.01	*TMEM132A*	0.01
GPN1	0.01		

Supplementary Table 4: The top ten up- (the left) and downregulated (the right) proteins in *CPTP*-knockdown cells.

Gene Symbol	Abundance Ratio	Gene Symbol	Abundance Ratio
NAT1	11.1	*DNER*	0.000962742
TRAM1	3.235691574	*TGFBI*	0.042735043
H2AFV	2.427950311	*PTPRF*	0.095238095
UROS	1.916450319	*KRT19*	0.230769231
PTGIS	1.80952381	*UBE2C*	0.262048193
PNKP	1.698533967	*TGM2*	0.292929293
SUB1	1.565560166	*MAP1LC3B*	0.391143911
CALD1	1.460713049	*EID2*	0.415019763
NUCKS1	1.373786408	*GATM*	0.452380952
SCD	1.037488708	*PRKD2*	0.51

Supplementary Table 5: Correlation between Sp1 expression and clinicopathological characteristics for PC patients.

	variables	Sp1 expression		total	χ^2	p value [a]
		low	high			
Age (year)					0.134	0.714
	<60	19	9	28		
	≥60	28	16	44		
Sex					0.547	0.46
	Female	20	8	28		
	male	29	17	46		
Grade					4.175	0.041*
	I/II	37	13	50		
	III	12	12	24		
T stage					0.309	0.578
	T1/T2	43	23	66		
	T3	6	2	8		
N stage					0.536	0.464
	N0	34	13	47		
	N1	14	8	22		
M stage					4.898	0.027*
	M0	47	20	67		
	M1	2	5	7		
TNM stage					0.557	0.455
	I/II	28	12	40		
	IV	21	13	34		
Tumor size					0.064	0.8
	<4 cm	22	12	34		
	≥4 cm	27	13	40		

* $P < 0.05$; ** $P < 0.01$

a Chi-squared test results

Supplementary Table 6: Correlation between Sp3 expression and clinicopathological characteristics for PC patients.

	variables	Sp3 expression		total	χ^2	p value [a]
		low	high			
Age (year)					0.700	0.403
	<60	15	12	27		
	≥60	19	23	42		
Sex					0.152	0.697
	Female	15	13	28		
	male	21	22	43		
Grade					9.982	0.002**
	I/II	31	18	49		
	III	5	17	22		
T stage					5.265	0.022*
	T1/T2	35	28	63		
	T3	1	7	8		
N stage					3.033	0.082
	N0	26	18	44		
	N1	8	14	22		
M stage					4.121	0.042*
	M0	35	29	64		
	M1	1	6	7		
TNM stage					8.791	0.003**
	I/II	25	12	37		
	IV	11	23	34		
Tumor size					0.012	0.914
	<4 cm	16	16	32		
	≥4 cm	20	19	39		

* $P < 0.05$; ** $P < 0.01$

a Chi-squared test results

Fig. S1 Expression levels of CPTP were detected by Western blot analysis. Ctrl, empty vector control; OE, overexpression; sh, short hairpin; NC, negative control.

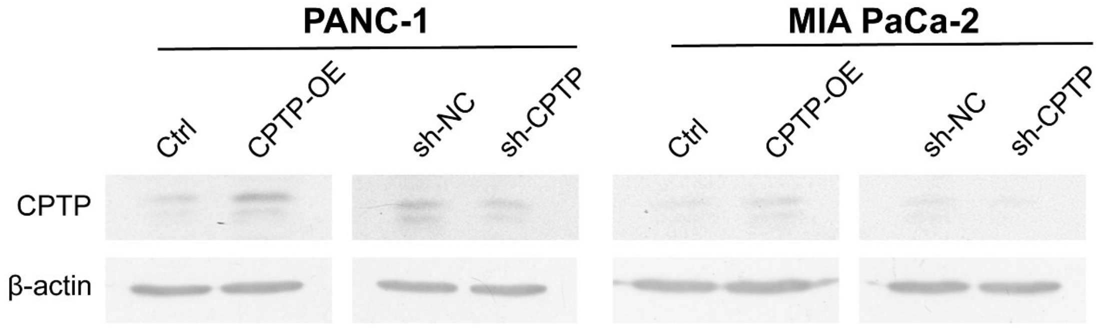

Fig. S2 Effects of *CPTP* on sphingolipid metabolites in the PANC-1 cell lines. Effects of overexpression and knockdown of *CPTP* on sphingomyelin in the PANC-1 cell line. *CPTP*, ceramide-1-phosphate transfer protein. A-B. Sphingomyelin (SM). C-D. Sphingosine (Sph). E-F. Lyso-phosphatidylcholines. *P < 0.05, **P < 0.01, ***P < 0.001.

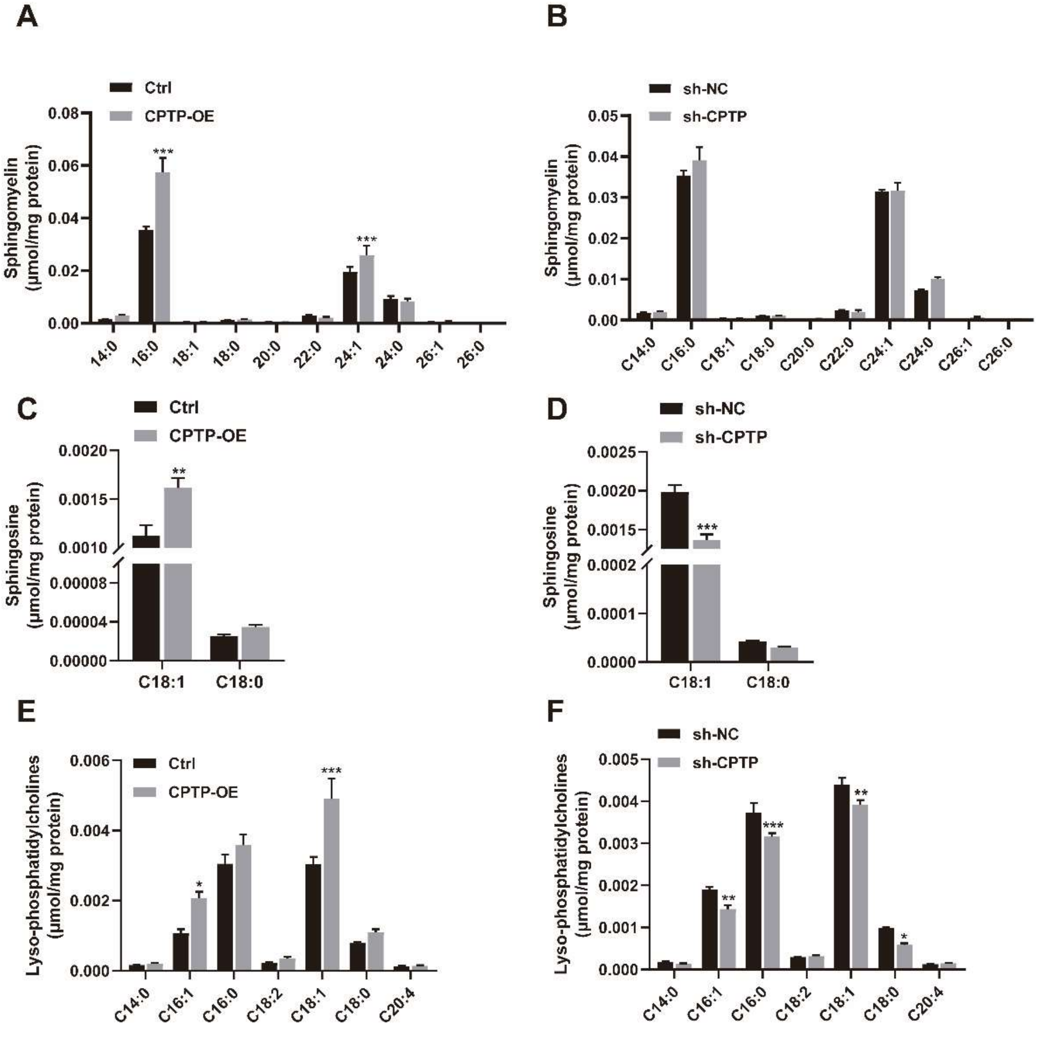

Fig. S3 Transcriptome sequencing analysis of *CPTP* following overexpression or knockdown in the PANC-1 cell lines. The distribution of differentially expressed genes in *CPTP* (A) overexpressing or (B) knockdown cells are shown using a volcano plot. Gene Ontology analysis of the differentially expressed genes for *CPTP* (C) overexpression or (D) knockdown in the PANC-1 cell lines. The top 10 enriched biological processes are shown.

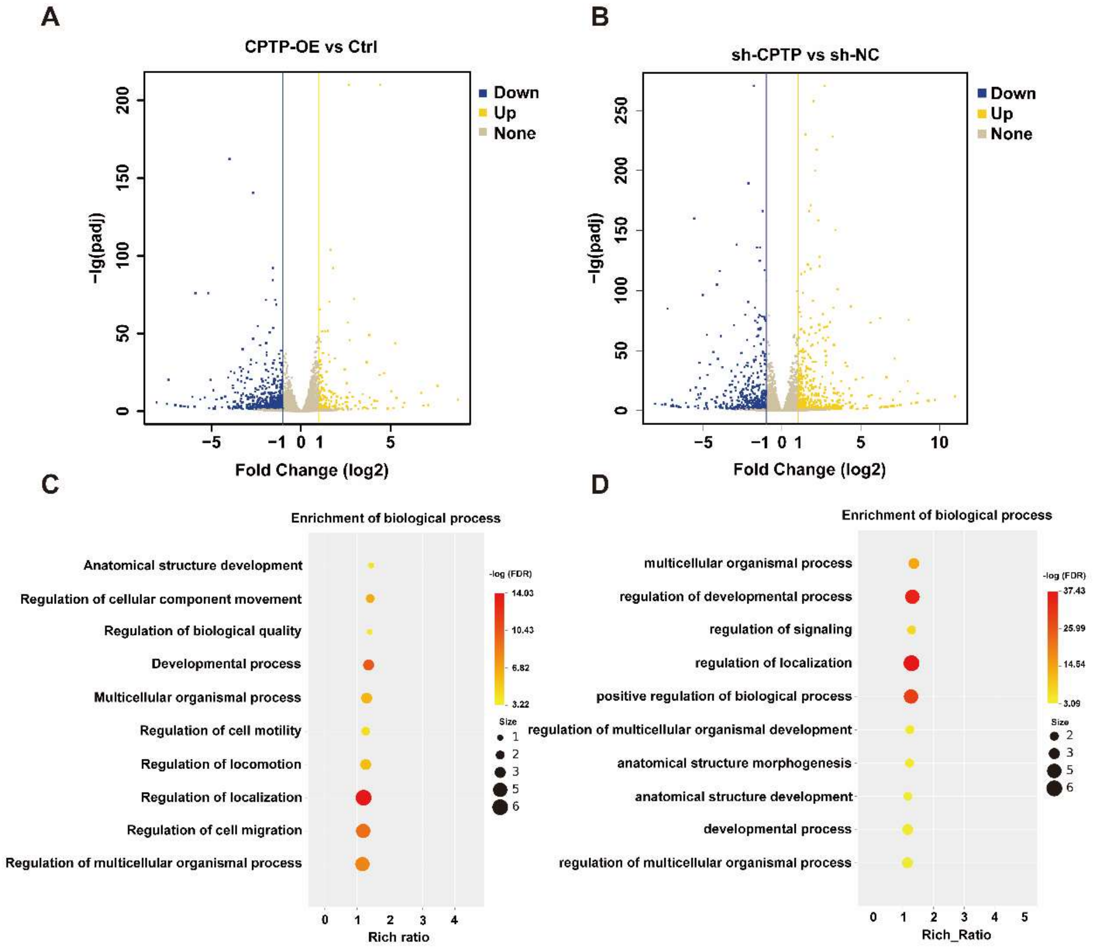

Fig. S4 Inhibition of PI4KA-mediated AKT phosphorylation represses cell proliferation, colony formation, cell migration and invasion in PANC-1 cells with *CPTP* overexpression. *CPTP*-overexpression PC cells (PANC-1) were grown to 40~60% confluence, and then recharged with fresh medium. GSK-A1 was added into the cells reaching a final concentration of 100 nM. After 24 h, cell proliferation (A), colony formation ability (B-C), cell migration and invasion (D-E) were assessed using CCK-8 assay, colony formation assays and Transwell assay. Effect of AKT phosphorylation was assessed using Western blot analysis (F). Vehicle, 0.1% DMSO. *P < 0.05, **P < 0.01, ***P < 0.001.

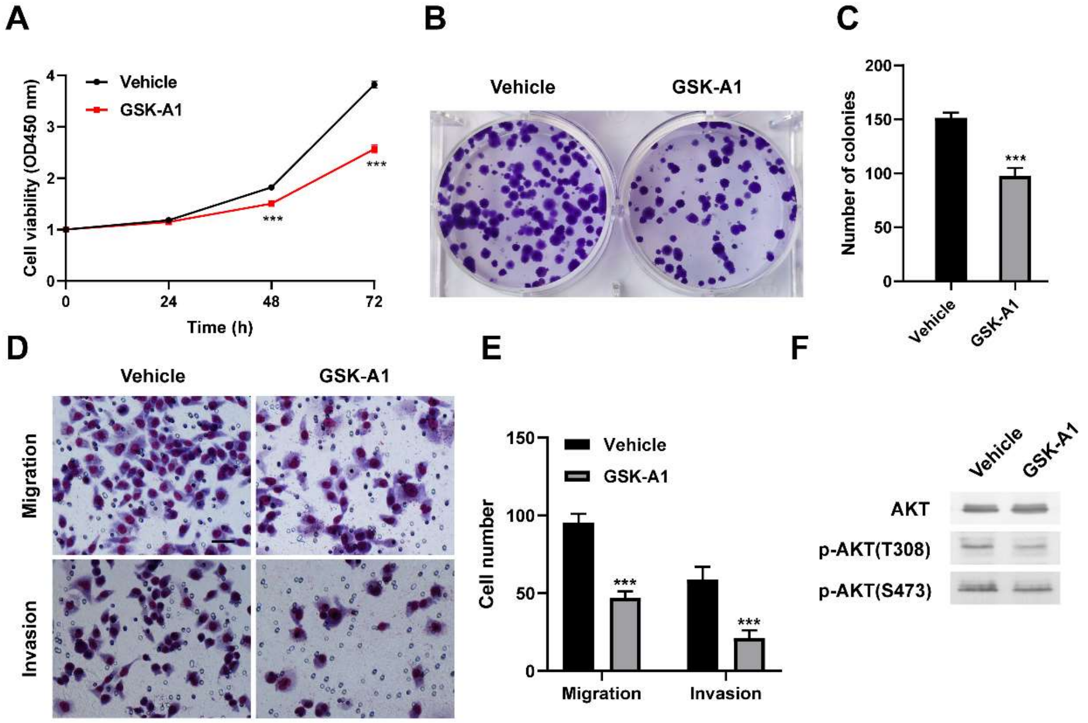

Fig. S5 Characterazation of transcriptional start sites（TSS）of human *CPTP*. A. Genomic structure of human *CPTP* in contig NC_000001.11. 5’-RACE amplification was performed using outer and inner primers in adaptor and gene-specific primers RA-1 and RA-2. B. Agarose gel electrophoresis of 5’ RACE amplification products for human *CPTP*. C. Sequence analysis indicates position of three transcriptional start sites of human *CPTP*.

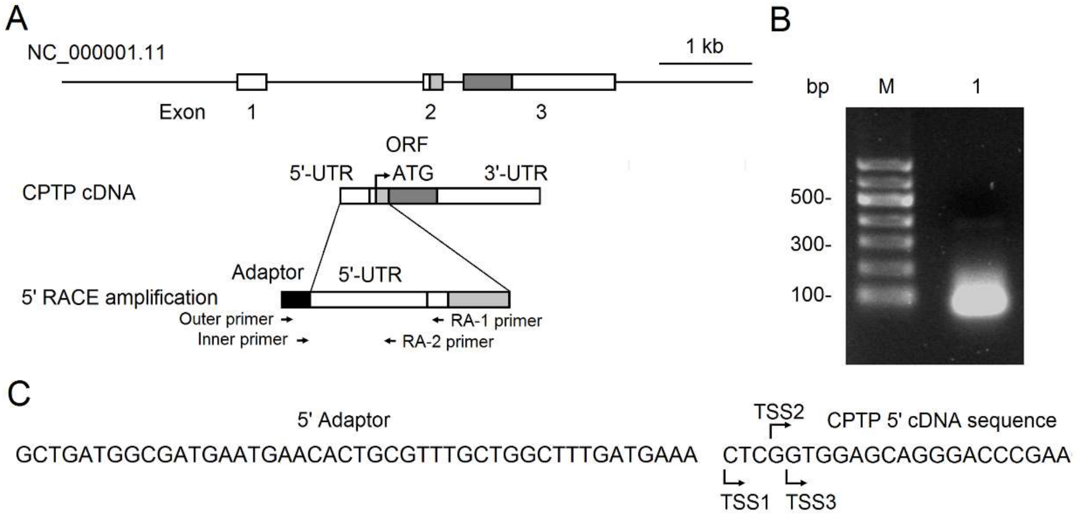

Fig. S6 High GC content upstream of *CPTP*. (A) GC content of the upstream CpG island region of CPTP. (B) Nucleotides in the basal promoter of *CPTP*. The two Sp1/Sp3 binding sites are shown in bold.

A

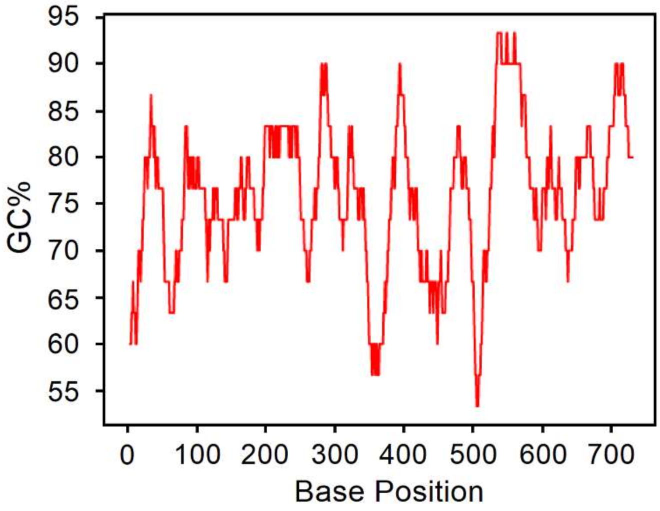

B

-454 AAGGGCGTGACTCTGATCTCAGGCATCGTCTCCGCCGCGC
-414 TCCCGGACCCGCGAGGCCCGCCTGCGGTGATGCACTGCGC
-374 AGGCGCAACCACCTCGCTGCAACTTCCGGTGCGCTAGCCG
-334 GAAACGCGGGTCGGGGCGCCGGGCCTCCTGATTGGGCAGC
-294 ATCCAACCAATC**AGGGCGGCGG**GCGAGGGCGGGGCG**AGGG**
-254 **CGGGGC**GGTGGGCGGGGACGGGGCCCGCACGGCGGCTACG
-214 GCC
